# Early-life formula feeding is associated with infant gut microbiota alterations and an increased antibiotic resistance load

**DOI:** 10.1093/ajcn/nqab353

**Published:** 2021-10-22

**Authors:** Katariina M M Pärnänen, Jenni Hultman, Melina Markkanen, Reetta Satokari, Samuli Rautava, Regina Lamendella, Justin Wright, Christopher J McLimans, Shannon L Kelleher, Marko P Virta

**Affiliations:** Department of Microbiology, University of Helsinki, Helsinki, Finland; Department of Microbiology, University of Helsinki, Helsinki, Finland; Department of Microbiology, University of Helsinki, Helsinki, Finland; Human Microbiome Research Program, Faculty of Medicine, University of Helsinki, Helsinki, Finland; Children's Hospital, University of Helsinki and Helsinki University Hospital, Helsinki, Finland; Department of Biology, Juniata College, Huntingdon, PA, USA; Wright Labs LLC, Huntingdon, PA, USA; Wright Labs LLC, Huntingdon, PA, USA; Department of Biomedical and Nutritional Sciences, University of Massachusetts Lowell, Lowell, MA, USA; Department of Cellular and Molecular Physiology, Penn State Hershey College of Medicine, Hershey, PA, USA; Department of Microbiology, University of Helsinki, Helsinki, Finland

**Keywords:** antibiotic resistance, neonate, infant formula, metagenomics, bioinformatics, microbiome, human milk, milk fortifier, pediatrics

## Abstract

**Background:**

Infants are at a high risk of acquiring fatal infections, and their treatment relies on functioning antibiotics. Antibiotic resistance genes (ARGs) are present in high numbers in antibiotic-naive infants’ gut microbiomes, and infant mortality caused by resistant infections is high. The role of antibiotics in shaping the infant resistome has been studied, but there is limited knowledge on other factors that affect the antibiotic resistance burden of the infant gut.

**Objectives:**

Our objectives were to determine the impact of early exposure to formula on the ARG load in neonates and infants born either preterm or full term. Our hypotheses were that diet causes a selective pressure that influences the microbial community of the infant gut, and formula exposure would increase the abundance of taxa that carry ARGs.

**Methods:**

Cross-sectionally sampled gut metagenomes of 46 neonates were used to build a generalized linear model to determine the impact of diet on ARG loads in neonates. The model was cross-validated using neonate metagenomes gathered from public databases using our custom statistical pipeline for cross-validation.

**Results:**

Formula-fed neonates had higher relative abundances of opportunistic pathogens such as *Staphylococcus aureus, Staphylococcus epidermidis, Klebsiella pneumoniae, Klebsiella oxytoca*, and *Clostridioides difficile*. The relative abundance of ARGs carried by gut bacteria was 69% higher in the formula-receiving group (fold change, 1.69; 95% CI: 1.12–2.55; *P* = 0.013; *n* = 180) compared to exclusively human milk–fed infants. The formula-fed infants also had significantly less typical infant bacteria, such as Bifidobacteria, that have potential health benefits.

**Conclusions:**

The novel finding that formula exposure is correlated with a higher neonatal ARG burden lays the foundation that clinicians should consider feeding mode in addition to antibiotic use during the first months of life to minimize the proliferation of antibiotic-resistant gut bacteria in infants.

## Introduction

Antibiotic-resistant pathogenic bacteria cause approximately 214,000 neonatal deaths annually (1). Thus, understanding factors that affect this vulnerable group's resistance load is crucial. Previously, it was presumed that antibiotic use plays the most significant role in the rise of antibiotic-resistant bacteria (ARB) in health-care settings. However, the transmission of ARB instead of antibiotic use has been suggested to play a major role in global clinical resistance (2).

Antibiotic use has a well-established role in shaping the resistome of infants. Antibiotic use affects the resistome in an antibiotic-specific way as bacteria resistant to the given antibiotic increase. Consequently, the abundance of the antibiotic resistance genes (ARGs) carried by the resistant strains increases simultaneously (3). It is known that the number and extent of antibiotic treatments in infancy affect ARG abundance (3, 4).

Infants have greater concentrations of ARGs in their gut microbiota than adults, even without being exposed to antibiotics (3, 5–7). Therefore, the transmission of ARB and other selective factors besides antibiotic treatment might drive ARG enrichment in neonates and infants. However, there is limited knowledge on the effect of selective pressure–causing agents other than antibiotics on resistance loads in neonates and infants. Mobile genetic elements (MGEs) transmit ARGs between bacteria, possibly spreading ARGs in the infant gut microbiota, and ARGs can be vertically transmitted from the mother or acquired from the hospital environment (8–12). Consequently, to reduce infant mortality caused by antibiotic-resistant bacteria, it is critical to reduce the chances of transmission and find ways to modify the gut environment to be less favorable for colonization by resistant strains.

Feed type can have a substantial effect on the microbiota, and previous studies suggest that diet can shift the abundance of specific ARGs in the infant gut (5, 6, 13–15). However, the magnitude of the impact of infant diet on the proportion of resistant and multiresistant bacteria in the gut, referred to here as the resistance load, is currently unknown.

We hypothesized that formula exposure, which influences the infant gut microbiota, would also cause variation in the average resistance load (5, 13, 14). We collected metagenomic data from cross-sectional fecal samples taken at the age of 7 to 36 days from 46 infants born prematurely between 27 and 36 weeks of gestation. The average resistance load was approximated by calculating the relative number of ARGs/copy of 16S RNA gene. The collected cohort was used as a train set to construct a generalized linear model (GLM) that could explain infant antibiotic resistance loads. The GLM was cross-validated with a custom statistical pipeline using an external test set comprised of publicly available neonatal gut microbiota metagenomes (3, 5, 15–17).

## Methods

### Description of the study cohorts

**FIGURE 1:**
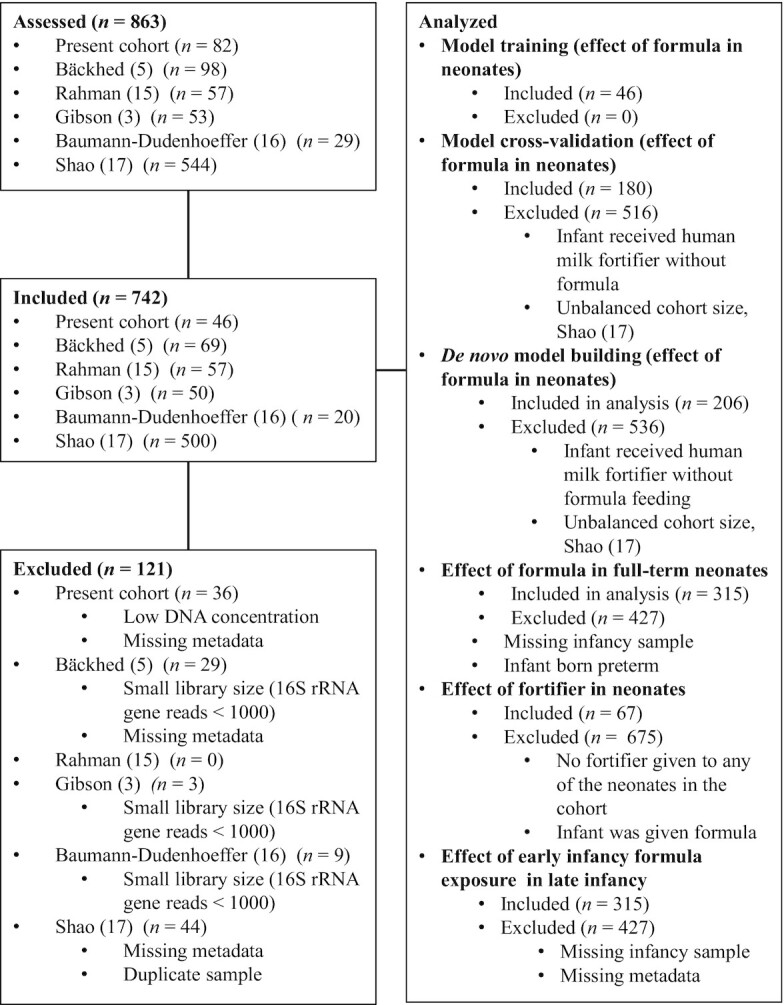
Participant flowchart outlining the participants included in the different analyses and criteria for exclusion. Abbreviations: rRNA, ribosomal RNA.

A total of 46 infants born prematurely between 26–37 weeks of gestation were included in phase 1 of the present study. Subjects were selected from a set of infants born at Penn State Hershey Medical Center (PSHMC) or transferred to the PSHMC neonatal intensive care unit (NICU) within 72 hours of birth (*n* = 82). The exclusion criteria were: inadequate DNA concentrations (less than 20 ng of total DNA) and missing clinical information (**Figure 1**). We collected extensive metadata on infant diet during the first month of life to investigate the effects of formula and fortifier exposure on the ARG load. We aimed to balance major clinical parameters between formula-fed infants and infants who did not receive any formula in their diet (**Table 1**). Infants were sampled approximately 2 weeks after antibiotic treatment, and the antibiotic treatment duration was short (0–2 days) to minimize the effect of antibiotics. Hospital personnel and sample collectors were not blinded for infant diet. Fecal samples were collected at less than 36 days of age, and Illumina NextSeq sequencing was performed at the Institute of Biotechnology (University of Helsinki).

**TABLE 1:** Clinical data for the study population for infants by formula exposure^1^

	Not formula-fed, *n* = 25	Any formula feeding, *n* = 21	Difference in means between groups^2^
Infant antibiotic treatment	18	12	—
Infant amp + gent treatment	15	11	—
Infant amp + gent + van treatment	3	1	—
Duration of infant antibiotic treatment, days, median (IQR)	2 (0–2)	2 (0–2)	—
Maternal antibiotic treatment	15	10	—
Cesarean delivery	14	15	—
Vaginal delivery	11	6	—
Weight at time of sampling, kg, median (IQR)	1.580 (1.360–1.980)	2.25 (2.060–2.400)	—^3^
Sex female	9	10	—^3^
Sex male	16	11	—
Infant age, days, median (IQR)	17.00 (14.00–17.00)	17.00 (14.00–17.00)	—^3^
Fortifier	20	0	—^3^
Breast milk only	5	0	—
Gestational age, weeks, median (IQR)	31.43 (29.86–32.86)	33.86 (33.43–34.00)	—^3^
Infant infection	4	2	—
Necrotizing enterocolitis	0	0	—
Prolonged rupture of membranes	9	3	—
No group B *Streptococcus*	9	14	—
Group B *Streptococcus*	6	2	—
Unknown group B *Streptococcus*	10	5	—
Maternal diabetes	2	2	—
Maternal preeclampsia	3	10	—^3^
Length of stay in hospital, days, median (IQR)	38 (26–61)	21 (18–24)	—^3^

1 *n* = 46. The table shows the major clinical parameters. Full clinical data are shown in Supplemental Data 1. Abbreviations: amp, ampicillin; gent, gentamycin; van, vancomycin.

2 The column entitled “Difference between groups” indicates instances where the groups were not balanced (Student's *t*-test).

^3^Significant difference between groups

Twenty-one infants were fed a commercial infant formula (Neosure), 20 were fed mother's milk with human milk fortifier (Similac), and 5 were fed only human milk (mother or donor). The infants born at <32 weeks received fortifier. Infants born at >32 weeks were fed formula per standard hospital feeding protocols for infants born prematurely. Thirty of the infants received antibiotics. In cases where the infant received antibiotics, fecal samples were collected approximately 2 weeks after the termination of antibiotic treatment to eliminate antibiotic therapy's potential confounding effect.

To obtain more samples to study the variables affecting the ARG load in the neonate gut, we collected a meta-analysis in addition to the original cohort of 46 infants. Literature searches were performed using the keywords “metagenomics” AND “infant,” “preterm” OR “newborn” OR “neonate.” We included all available publications where neonatal gut samples (with samples taken between 0 and 36 days of age) had been shotgun metagenome sequenced using NextSeq or HiSeq (Illumina) with read lengths from 100 to 250 base pairs. Only sequencing data sets with 20 or more individually sampled infants were taken. Other requirements were that data for gestational age, age at sampling, delivery mode, diet until the day of sampling, and antibiotic treatment of the infant were available. After filtering out data sets that did not fulfill the qualifications, 5 data sets were included in the meta-analysis (3, 5, 15–17). The data were downloaded in fastq format, and in cases where infants were sampled more than once, we used only the first sample. Only samples with more than 1000 16S ribosomal RNA (rRNA) reads identified using Metaxa2 were taken to the meta-analysis (18). In total, the meta-analysis cohorts had 696 neonates. The participant flowchart for each of the different analyses is depicted in Figure 1.

### Experimental design

**FIGURE 2:**
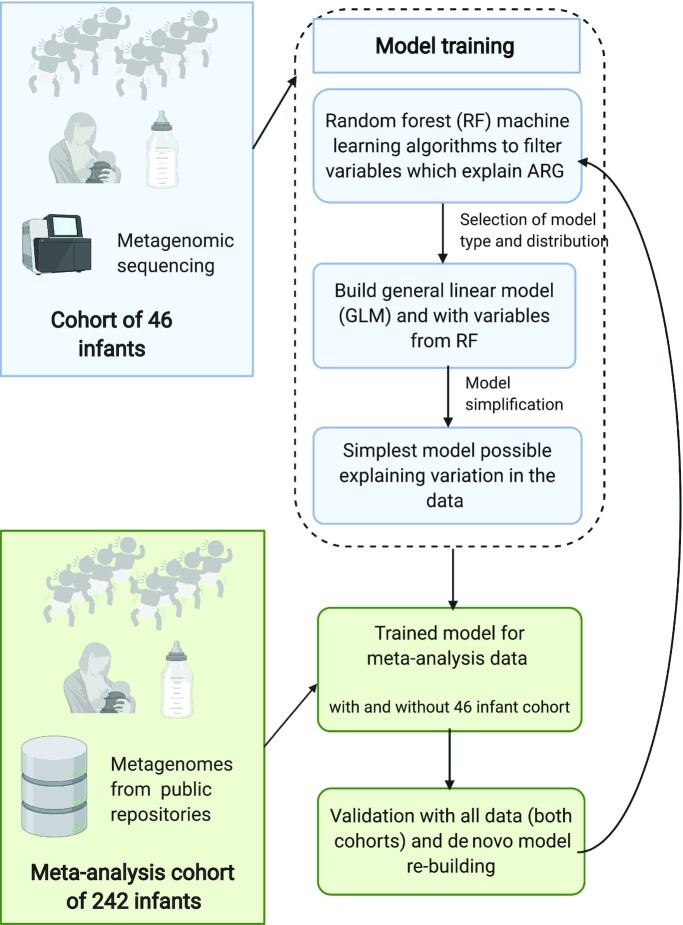
Flowchart of analysis methods, describing the included cohorts and the statistical analysis pipeline devised for this study. The pipeline was used to ensure that the effect of diet could be validated in several points of the analysis and that suitable distributions and model types are applied to the data. Blue boxes describe steps in preliminary analysis with the training set of 46 infants, and green boxes steps taken in model testing and cross-validation with meta-analysis cohorts. Abbreviations: ARG, antibiotic resistance gene.

Our a priori hypothesis was that diet impacts the gut microbiota of preterm infants and that infants fed formula would have a higher ARG load than infants fed only human milk, due to the changes in the community composition. To study the effect of formula feeding, we drafted a statistical analysis pipeline, including several steps to ensure model quality and the robustness of the results (**Figure 2**).

As the first step in the statistical analysis pipeline outlined in Figure 2, we used a random forest (RF) machine-learning algorithm to identify explanatory variables linked to ARG load in the preterm infant feces for ARG load model training. We used RF variable importance to prefilter clinical parameters and metadata to select which clinical variables we should use in the subsequent model building.

Secondly, once we determined that the responses were linear, we used gamma-distributed generalized linear models. We chose this distribution because the data were ARG counts normalized with 16S rRNA gene count, and thus were unsuitable for negative binomial or quasi-Poisson distributions, and because the data had overdispersed variances violating the normality assumption. GLMs are a potent tool for hypothesis testing but thus far have been used in only a limited number of microbiome studies (4, 19). After the GLM distribution was selected, we used the variables that could potentially affect ARG load to train the model using the preterm cohort. Explanatory variables that did not significantly explain variance in the ARG loads or did not improve the model were dropped 1 at a time from the full model using Akaike information criterion (AIC) values and chi-squared tests (α = 0.05). Model training ultimately resulted in the simplest model possible explaining the variation in the data without overfitting the model (20).

Thirdly, we confirmed the applicability of the model built using data gathered for a meta-analysis as a test set. Lastly, we created a de novo model, following similar steps as for the train set, to confirm the association of formula with an increased ARG load even when we considered all data and all clinical variables.

### Ethical approval

The Institutional Review Board of Pennsylvania State University (IRB #35925) approved the study.

### Sample collection

Enrolment commenced in January 2014 and concluded in July 2017. Inclusion criteria were preterm infants born 26–37 weeks of gestation who were admitted to the Penn State Hershey NICU or transferred to the PSHMC NICU within 72 hours of birth. Exclusion criteria included the following: infants born at <26 or >37 weeks of gestation, infants born with major congenital anomalies (heart, gastrointestinal, renal, or respiratory tract), mothers known to use illicit drugs or abuse alcohol, or mothers with a history of depression requiring long-term psychotropic medication. We obtained written consent from all subjects’ parents within 48 hours of admission to the PSHMC NICU. Metadata on feed history, clinical course, and necrotizing enterocolitis (NEC) outcomes were collected electronically during the entire course of stay in the PSHMC NICU.

Fecal material was collected into sterile microcentrifuge tubes approximately 2 weeks after prophylactic antibiotics had been discontinued and enteral feeds were initiated. Samples were frozen at −80°C until analysis.

### DNA isolation and quantification

Fecal samples were collected and shipped to Wright Labs, LLC. Nucleic acid extractions were performed from approximately 0.25 g of feces using the Qiagen DNeasy PowerSoil DNA Isolation kit following the manufacturer's instructions (Qiagen). The lysing was performed using the Disruptor Genie cell disruptor (Scientific Industries). Genomic DNA was eluted in 50 μL of 10 mM Tris. Subsequent quantification was performed using the Qubit 2.0 Fluorometer (Life Technologies) using the double-stranded DNA high-sensitivity assay.

### Metagenomic sequencing

The Nextera XT library preparation kit (Illumina) with the manufacturer's standard protocol was used for library preparation. The library was paired-end sequenced using 1 NextSeq run targeting an average of 10 million reads per sample. Sequencing and library preparation were done at the Institute of Biotechnology's sequencing services (University of Helsinki).

### Metagenomic analysis

We performed quality control using the FastQC and MultiQC programs (21, 22). The sequences were trimmed using Cutadapt version 1.10 with the options -m 1, -e 0.2, -O 15, and -q 20 to filter out adapters and low-quality reads (23). Filtered metagenomic sequence reads underwent subsequent microbial community profiling using the annotation software MetaPhlAn2 version 2.6.0 with default settings (24). A merged abundance table was created using the MetaPhlAn2 ’utils’ script. The merged abundance table was edited to only include taxa, which MetaPhlAn2 identified up to species level.

The 16S rRNA gene sequences were identified, extracted, and quantified using Metaxa2 version 2.6.0 in paired-end mode with default settings (18). The 16S rRNA gene sequence reads were classified using the mothur version v.1.40.5 “classify. seqs” command with SILVA version 123 as the reference database, with the options cutoff set at 60, probs set as F, and processors set at 8 (25, 26). A custom Unix script was used to create an OTU (operational taxonomic unit) table based on the classifications. Samples with less than 1000 reads mapping to the 16S rRNA gene were filtered out. Metabolic genes were annotated using the HUMAnN2 (HMP Unified Metabolic Analysis Network) pipeline with default settings and with enzyme categories for gene annotation and grouping (27).

We used Bowtie2 for mapping reads to ARG and MGE databases with the options -D 20, -R 3, -N 1, -L 20, and -i S,1,0.50 28). Following annotation, we used SAMtools version 1.4 was to filter and quantify observed ARG and MGE annotations within each sample (29). If both reads mapped to the same gene, we counted the read as 1 match, and if the reads mapped to different genes, both were counted as hits to the respective gene. We used the ResFinder database version 2.1 to search for acquired ARGs and an MGE database, including genes related to or annotated as IS (insertion sequence), ISCR (insertion sequence common region), *intI1, int2, istA, istB, qacEdelta, tniA, tniB, tnpA*, and Tn*916* transposons (6, 30). We searched for plasmid associated genes using the PlasmidFinder database (31).

### ARG and MGE count normalization

The Bowtie2 counts for ARGs and MGEs were normalized to the length of each respective gene. Normalized gene counts were then further normalized to the number of bacterial 16S rRNA gene reads obtained from Metaxa2 output, divided by the 16S rRNA gene's length (18). These normalization steps yield an approximation for the number of genes per 16S rRNA sequence for each resistance gene while avoiding bias due to the differential length of the resistance genes. We chose the 16S rRNA gene for normalization instead of library size to account for variation in nonbacterial DNA content in the samples, as explained in Bengtsson-Palme et al. (32). The 16S rRNA gene normalization allows us to approximate the number of resistance genes carried by bacteria, presuming that the copy number of 16S rRNA genes is similar in studied bacterial populations. The 16S rRNA gene-normalized values were used in all downstream analyses. However, we also compared unnormalized data and library size normalized data to validate our choice of normalization, and these yielded similar results to 16S rRNA normalized values (**Supplemental Results**).

In addition to the 16S rRNA gene, we used the single copy *rpoB* gene for alternative normalization. HMMER3 and the *rpoB* Hidden Markow model retrieved from Pfam database were used to count hits (33, 34). The “hmmsearch” command was run with an e^–02^ threshold on sequences translated to amino acids in all 6 reading frames. Length normalization was done in the same way as for the 16S rRNA gene. Statistical modeling for *rpoB* gene counts was done using GLMs and the quasi-Poisson distribution suitable for overdispersed count data (Supplemental Results).

### Meta-analysis

We collected metadata for the samples from the National Center for Biotechnology Information Sequence Read Archive or related publications’ supplementary materials (3, 5, 15–17). The metadata used in the meta-analysis are shown in Supplemental Data 3. We also ran all models without the firstborn twin to confirm estimates were not affected by twin pairs, since some of the data sets included twins. We interpreted studies that specified the infants were healthy or that pregnancies were normal as indicating that subjects did not have NEC or preeclampsia. We also analyzed the effects of other clinical parameters, such as maternal diabetes, infant infection status other than NEC, and antibiotic treatment duration, for those subcohorts where data were available. However, they did not significantly improve the models.

We did a preliminary analysis on the long-term effects of being fed formula using data from Bäckhed et al. (5), Baumann-Dudenhoeffer et al. (16), and Shao et al. (17). **Supplemental Note 2** lists the European Nucleotide Archive accession numbers for the infants included in the longitudinal analyses from the Bäckhed et al. (5) and Shao et al. (17) studies. The accession numbers of the Baumann-Dudenhoeffer et al. (16) study are in **Supplemental Data 3**.

GLMs confirmed the independence of the results of the 5 different cohorts and sequencing platform and library preparation method combinations, as including these explanatory variables in the model did not improve the fit (**Supplemental Data 4**).

We postulated that infants fed formula in addition to fortified human milk would display similar effects as infants fed formula in addition to unfortified human milk. We also assumed that infants fed fortified human milk might differ from infants receiving only human milk. Therefore, we excluded all infants fed fortified human milk that did not receive formula and compared human milk−exclusive diets and formula-containing diets (*n* = 206). We separately analyzed the effects of human milk supplemented with fortifier compared to an exclusively human milk–based diet. In Bäckhed et al. (5), gestational ages were between 39.3 and 41 weeks. Gestational ages were not reported separately for each infant, and we used an approximate gestational age of 40 weeks for all infants in this cohort. ARG annotation and quantification, as well as Metaxa2 and MetaPhlAn2 community profiling, were conducted for the obtained meta-analysis data set as described above (18, 24).

### Statistical analysis: model type selection and model training

All statistical analyses were performed in R version 3.6.1 (R Studio). MetaPhlAn2, Metaxa2, and HUMAnN2 annotation; ARG and MGE mapping results; taxonomy; and metadata files were compiled into individual data objects in phyloseq version 1.28.0 (18, 24, 27, 35). All custom R codes are available from https://github.com/KatariinaParnanen. All figures were plotted with ggplot2 version 3.1.1 (36). The flow of the statistical analysis pipeline is outlined in Figure 2.

An RF analysis was performed using the caret package version 6.0–84 with training control using the “trainControl” command with the option method “cv” and model fitting with “train” command with the option method “rf,” and the importance of the variable was computed using the “varImp” command (37). All variables in the metadata were used in the RF ANOVA importance (for the full list of available variables, see Supplemental Data 1) to model the outcome variable relative abundance of ARGs normalized by gene lengths and 16S rRNA gene counts. Weight, which correlated with both age of the infant and gestational age, and the type of maternal antibiotics, where inadequate numbers of mothers received the same antibiotic, were omitted from the analysis.

We compared different statistical model types to select the best option for modeling the ARG/16S rRNA gene ratio in the individuals from the 46-infant cohort. We examined the data for signs of overdispersion. Because the variance for untransformed data was much larger than the mean indicating overdispersion, we excluded models with normality assumptions. The data had positive continuous values, and the responses seemed to be linear. Therefore, we considered log-transformed normal and gamma distributions as possible distributions for modeling the data. Gamma distributions captured more of the variance than log-transformed normal distributions. Thus, we chose the GLM with gamma distribution and a natural logarithmic link function. The GLM models were fitted with the “glm” command using the option family “gamma” (link = “log”).

Model training for relative ARG abundance was performed with the “step” function, relying on AICs and using chi-squared tests by dropping out 1 variable or interaction term at a time and checking whether the simplified model performed significantly worse than the model with more explanatory factors. Diet was coded as any formula (Formula), any fortifier (Fortifier), or no formula or fortifier (Breast milk). We used the resulting simplified model from model training to analyze the meta-analysis cohorts without the trainset of 46 infants (*n =* 180) and with the trainset cohort to investigate whether the model estimates change. We also reperformed model selection with all the clinical metadata and all the infants (*n* = 206) to examine whether formula was retained in the rebuilt model that used all the meta-analysis data.

### Statistical analysis for meta-analysis cohorts

For the meta-analysis, we explored generalized linear mixed-effect models to investigate whether we should include random effects caused by the study design, as well as sequencing and library construction methods in the different meta-analysis cohorts, in the model. However, the models indicated that there was insufficient variation between cohorts to warrant random effects. Models with the study as a random effect resulted in singularity, indicating that the random structure was too complicated. The random structure could not be simplified further, meaning there were no study-dependent structures in the variance. Thus, we chose gamma-distributed GLM without random effects to study the relative ARG abundances.

To analyze the effect of formula on the relative ARG abundance, we compared formula-fed infants to infants who were exclusively fed human milk. To this end, we excluded all infants fed fortified human milk who did not receive any formula, which allowed for the analysis of 206 neonates (or 180 neonates after excluding the infants from the train set). Because Bäckhed et al. (5) did not report antibiotic use for each infant individually but only stated that 2 of the 100 infants were treated with antibiotics, these data were excluded from model validation on antibiotics’ effects in the meta-analysis.

We separately analyzed the effect of human milk supplemented with fortifier on the relative ARG abundances compared to an exclusively human milk–based diet. Power calculations to estimate effect sizes and sample sizes for a given effect size were performed using the “modelPower” and “modelEffectSizes” commands in the lmSupport package version 2.9.13 (38).

We also explored log-linear models, but they underperformed compared to the gamma-distributed model, with AIC values of around 700 compared to 40 and *R^2^* values of 0.16 compared to pseudo-*R^2^* values of 0.6 of the GLMs. Both metrics indicated worse performance for log-linear models than for GLMs.

DESeq2 version 1.24.0 was used to analyze differentially abundant taxa and genes (39). MetaPhlAn2 results were transformed to counts for DESEq2 analysis by multiplying the total 16S rRNA gene counts in each sample obtained from Metaxa2 with the relative abundance values for each sample from MetaPhlAn2. The transformed count data allow an approximation of the differences in the abundance of a bacterial species relative to all bacterial species between 2 groups of interest, such as infants given formula and breast-fed infants. Gene abundances were transformed by multiplying by 50,000 and rounded to integers, resulting in normalized values that consider variation in the genes’ lengths and the 16S rRNA gene counts in different samples. Using this transformation, the difference between a detected and an undetected gene is approximately 1.7-fold. A pseudo count of plus 1 was added to all samples to enable comparisons between detected and undetected genes. We tested several different options for transformation, but the results did not differ.

Principal coordinate analyses for the taxonomic profiles, metabolic pathways, ARGs, and MGEs were performed using the “ordinate” command from the phyloseq package (35). Distances between samples were calculated using the Horn-Morisita similarity index and oriented using principal coordinate analysis (40). Permutational multivariate analysis of variance (PERMANOVA) between different groups was performed with adonis from the vegan package version 2.5–5 using 9999 permutations, and the resulting *P* values were corrected with the Benjamini-Hochberg procedure for multiple testing using the “p.adjust” command in R (41).

Distance matrices for species, ARGs, and MGEs were calculated using the Horn-Morisita similarity index with the “vegdist” command in the vegan package and compared to observe whether there were correlations between microbial community and the ARGs and MGEs (40). Comparisons were performed using a Mantel's test from the vegan package with the option method “Kendal” (41). Shannon and Simpson diversities for taxonomic profiles, ARGs, and MGEs were calculated using the package vegan. Differences in the diversities were compared using an ANOVA and combined with Tukey's post hoc test from the “multcomp” R package to obtain adjusted *P* values for the pairwise comparisons. ARG abundances in samples dominated by different taxa were modeled with gamma-distributed GLMs, and Tukey's post hoc test from the “multcomp” R package was used to obtain adjusted *P* values for the pairwise comparisons.

## Results

### ARG load is affected by diet and gestational age in model built using train set

We performed initial model-building steps using the preterm cohort as a train set and followed our statistical analysis pipeline outlined in Figure 2. We first performed an RF analysis to screen those variables listed in Supplemental Data 1 with a possible effect on ARG load and, after selecting gamma distribution and generalized linear models as the appropriate analysis method, we proceeded to model building, dropping 1 variable at a time from the full model as described in Figure 2. The final model included gestational age, 16S rRNA counts, and formula.

**FIGURE 3:**
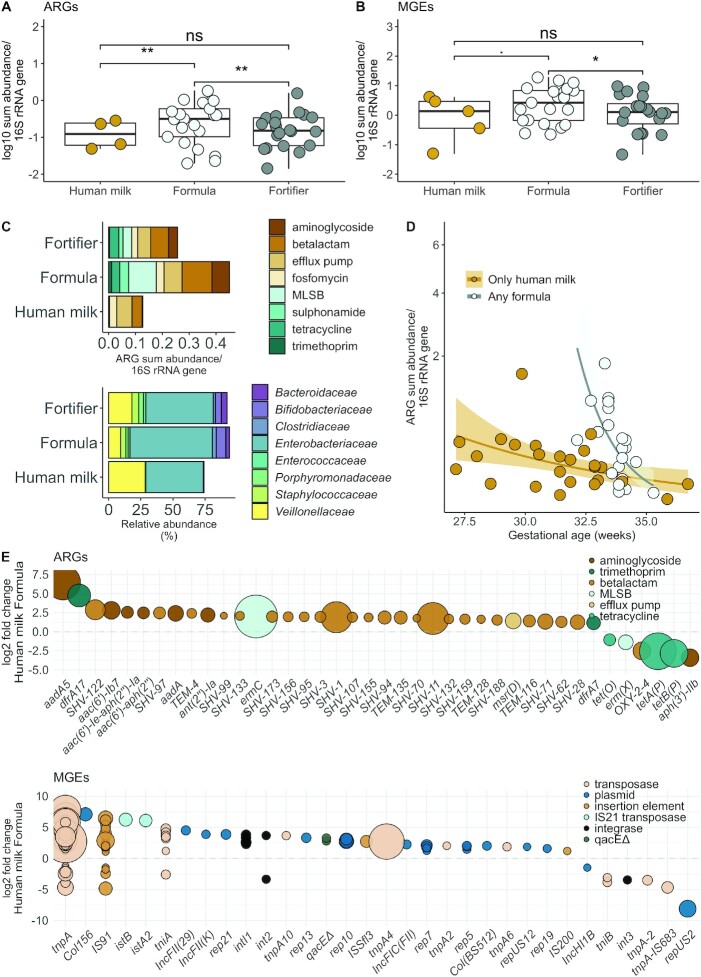
Effects of diet and gestational age on ARGs and MGE abundances in the preterm infant cohort. (A) Relative ARG and (B) relative MGE sum abundances by a diet containing either only human milk, any formula, or human milk and fortifier (*n* = 46). Infants fed any formula had higher ARG abundances (normalized to 16S rRNA counts) than infants fed only breast milk or fed milk supplemented with fortifier [gamma-distributed GLM adjusted with gestational age and 16S rRNA counts, fold changes 4.3 (95% CI: 1.61–11.56) and 3.6 (95% CI: 1.61–8.9), respectively; adjusted *P* < 0.01]. MGEs were significantly more abundant (normalized to 16S rRNA gene) in the infants fed any formula than in infants fed exclusively human milk (gamma-distributed GLM, fold change 2.07 (95% CI: 1.08–3.99; *P* < 0.05). (A and B) The *y* axis denotes log_10_ transformed ARG or MGE copies normalized by 16S rRNA gene copied counted from shotgun sequence data. Significance levels are denoted as follows: ** = 0.001–0.01; * = 0.01–0.05; . = 0.05–0.1; ns = 0.1–1. The boxplot hinges represent 25% and 75% percentiles and centerline the median. Notches are calculated with the formula median ± 1.58 × IQR/sqrt (*n*). (C) Differences between most abundant ARG classes and bacterial families. The *x* axis represents ARG copies normalized to 16S rRNA gene counts and percentage of bacterial family. (D) Effect of gestational age and formula on relative ARG abundances. The *x* axis represents gestational age in weeks. The *y* axis has been square root transformed. (E) Differentially abundant ARGs and MGEs using DESeq2 and an adjusted *P* value cutoff of 0.05 for the reported genes. Genes with positive values are more abundant in formula-fed infants. The larger the point size, the more abundant the gene is in the samples. Abbreviations: ARG, antibiotic resistance gene; GLM, generalized linear mode; MGE, mobile genetic element; MLSB, macrolide-lincosamide-streptogramin B; rRNA, ribosomal RNA.

We could not link antibiotic treatment of the infant or the mother to higher ARG loads [gamma-distributed GLM adjusted with diet and gestational age and 16S rRNA counts, fold changes 1.2 (*P =* 0.68) and 1.6 (*P* = 0.10), respectively]. However, our study was designed to eliminate the effect of antibiotic use since our interest was studying other effectors than antibiotics, since their effects have been studied in detail before, for example in Gasparrini et al. (4). Still, infants fed any formula had significantly higher ARG abundances (normalized to 16S rRNA counts) than infants fed only breast milk or fed milk supplemented with fortifier (gamma-distributed GLM adjusted with gestational age and 16S rRNA counts, fold changes 4.3 (95% CI: 1.61–11.56) and 3.6 (95% CI: 1.61–8.9), respectively; adjusted *P* values < 0.01; **Figure 3**A). However, infants fed fortifier were more premature than infants fed formula, as per hospital protocol, which causes a confounder. Additionally, we analyzed differences in ARG loads using a more specific diet description, including different feed sources (**Supplemental Data 2**).

We next determined whether there was an effect of fortifier supplementation on the ARG load. We observed no differences between infants fed fortifier compared to infants fed only breast milk (gamma-distributed GLM, Benjamini-Hochberg–adjusted *P* > 0.05; Figure 3A). Additionally, there were no differences between human milk–derived or bovine milk–derived fortifiers (gamma-distributed GLM, Benjamini-Hochberg–adjusted *P* > 0.05; Supplemental Data 2). These results suggest that in cases where infants require supplementary nutrition, the addition of fortifier to human milk might have less of an impact on the antibiotic resistance potential than switching to formula.

In our data set, MGEs were significantly more abundant (relative abundance normalized to 16S rRNA gene) in the infants fed any formula than in infants fed exclusively human milk (gamma-distributed GLM, fold change 2.07; 95% CI: 1.08–3.99; *P* < 0.05; Figure 3B). High numbers of MGEs and ARGs could suggest that resistance genes might be transferred between bacterial strains. Horizontal transfer of ARGs can also cause the emergence of new antibiotic-resistant strains of potentially clinically relevant pathogens (42). ARG classes and bacterial families also varied with infant diet (Figure 3C). The resistome and microbial community composition were significantly correlated and grouped by the dominant genus in the microbiota (Supplemental Results; Figure 1).

*Enterobacteriaceae* are known to harbor several mobile ARGs in their genomes; thus, we determined the effect of diet on the *Enterobacteriaceae* abundance. *Enterobacteriaceae* were more abundant in infants fed any formula than in infants who were fed human milk (quasibinomial GLM, approximately 3-fold higher abundance; gestational age as a cofactor, *P* < 0.05). These data are in line with a previous report indicating that formula increases the abundance of fecal *Enterobacteriaceae* in infants born preterm (43). There were no significant associations between the abundance of *Enterobacteriaceae* and other clinical parameters. However, gestational age tended to be inversely correlated with the *Enterobacteriaceae* abundance (quasibinomial GLM, 22% decrease/week of gestation; *P* < 0.1). Interestingly, *Enterobacteriaceae* have been linked to the onset of NEC, and both lower gestational age and formula feeding are well-established risk factors for NEC. Gestational age significantly affected the ARG load. Longer gestation translated to a lower ARG abundance (Figure 3D; gamma-distributed GLM, fold change, 0.72; 95% CI: 0.57–0.89; *P* < 0.001).

Several ARGs were significantly more abundant in formula-fed infants, including SHV genes, which encode the extended-spectrum beta-lactamase (ESBL) phenotype in *Klebsiella* (DESeq2: adjusted *P* < 0.05; Figure 3E). The relative abundance of SHV genes correlated with Klebsiella relative abundances (quasibinomial GLM, fold change for SHV of 4% per 1% more *Klebsiella*; 95% CI: 1.8%–6.7%; *P* = 0.002). Also, genes of all the studied MGE classes were more abundant in formula-fed infants than in human milk–fed infants (DESeq2; Figure 3E). The enriched genes included the integrase gene, *intI1*, which is part of the conserved region of class I integrons known to sometimes confer multidrug-resistant phenotypes in clinically relevant bacteria (44).

### Cross-validation corroborates that formula increases ARG load in neonates

We next sought to corroborate the impact of diet on the infant gut ARG load by testing our model on subjects from other metagenome cohorts. We included samples from 4 metagenomic studies. The participant flow chart for the subjects is shown in Figure 1. We excluded cohorts matching our literature reach that did not have metadata for gestational age, diet, antibiotic exposure, or infant age, and which included less than 20 infants or had an unbalanced cohort size compared to other cohorts, resulting in the inclusion of 4 cohorts. An additional fifth cohort by Shao et al. (17), a cohort excluded from model testing, was used to investigate the effect on formula feeding in full-term infants during infancy, and the results are described in Supplemental Results (17). The exclusion criteria for each study regarding preexisting maternal and infant health conditions and maternal drug and substance use are available in the original publications. A summary of clinical parameters from the neonates included in the meta-analysis is shown in **Table 2** and described in more detail in Supplemental Data 3 and**5**. Supplemental Data 4 shows the results of variable auto-correlations.

**TABLE 2:** Clinical data for the study population for infants by formula exposure in the meta-analysis cohorts^1^

	Human milk, *n* = 106	Any formula, *n* = 90	Difference in means between groups^2^
Infant antibiotic treatment	50	54	—
Infant ampicillin	49	54	—
Infant gentamycin	42	53	—^3^
Infant vancomycin	18	9	—
Infant cefotaxime	7	9	—
Infant clindamycin	0	2	—
Infant cefazolin	0	1	—
Infant ofloxacin	0	1	—
Infant meropenem	0	1	—
Infant ticarcillin clavulanate	2	1	—
No maternal antibiotic treatment	22	31	—
Maternal antibiotic treatment	34	40	—
Delivery mode cesarean	41	57	—
Delivery mode vaginal	65	33	—
16S rRNA gene counts median (IQR)	26,346 (7598–36,040)	38,820 (22,106–58,453)	—^3^
Sex female	59	53	—
Sex male	47	37	—
Infant age, days, median (IQR)	7.0 (2.25–14.172)	7.0 (4.00–10.75)	—
Fortifier^4^	16	8	—
Donor milk	2	2	—
Gestational age, weeks, median (IQR)	37 (28–40.0)	31 (29–37.75)	—
Necrotizing enterocolitis	3	7	—
Maternal preeclampsia	16	8	—

1 *n* = 196. The table shows the major clinical parameters by diet for the meta-analysis cohorts. Supplemental Data 3 has all collected metadata.

2 The column entitled “Difference between groups” indicates instances where the groups were not balanced (Student's *t*-test).

^3^Significant difference between groups

4 Infants who received fortifier but did not receive formula were excluded from analyses for the effect of formula making the *n* = 180.

**FIGURE 4:**
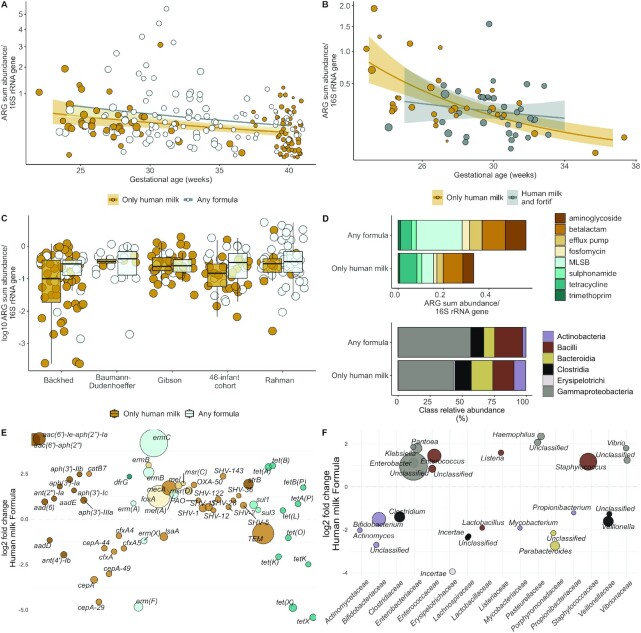
Effect of formula feeding on neonatal gut resistome in meta-analysis cohorts. (A) Differences in relative ARG sum abundance/16S rRNA gene copy numbers in infants only fed human milk or any formula. (*n* = 206; *n* = 180 for other cohorts; *n* = 26 for infants not fed fortifier in the train set cohort). Formula feeding was associated with an approximately 70% increase in the relative abundance of ARGs in the meta-analysis [*n* = 180; gamma-distributed GLM, fold change 1.69 (95% CI 1.12, 2.55); *P* = 0.013, adjusting for gestational age and 16S rRNA counts]. (B) Differences in relative ARG sum abundance/16S rRNA gene copy numbers in infants fed human milk or any fortifier (*n =* 67). Neither supplementation of the infant's own mother's milk with fortifier or donor milk had a significant effect on the ARG load (gamma-distributed GLM, *P* = 0.42 for fortifier; *P* = 0.91 for donor milk, adjusting for 16S rRNA counts and gestational age). (A and B) A regression line is fitted using gamma-distributed GLMs. The *y* axes have been square root transformed. Dot sizes depict infant ages, with larger dots being older infants. (C) Effect of formula on ARG load in meta-analysis data sets by cohort (*n* = 206). The boxplot hinges represent 25% and 75% percentiles and centerline the median. Notches are calculated with the formula median ± 1.58 × IQR/sqrt (*n*). (D) Differences between most abundant ARG and bacterial classes by diet in meta-analysis data sets. (E) Differentially abundant ARGs in infants with different diets using DESeq2 analysis and an adjusted *P* value cutoff of 0.05 for reported genes. Genes with positive values are more abundant in formula-fed infants, and genes with negative values are more abundant in exclusively human milk–fed infants. (F) Differentially abundant genera in infants with different diets using DESeq2 analysis and an adjusted *P* value cutoff of 0.05 for reported genera. Genes and genera with positive values are more abundant in formula-fed infants, and genes with negative values are more abundant in exclusively human milk–fed infants. (E and F) The larger the point size, the more abundant the gene is in the samples. Abbreviations: ARG, antibiotic resistance gene; GLM, generalized linear mode; MLSB, macrolide-lincosamide-streptogramin B; rRNA, ribosomal RNA.

We tested the model built with the train set (ARG load ∼16S rRNA counts + gestational age + formula) using the infants from the 4 other included meta-analysis cohorts (Figure 1). Infants with diets having any formula and exclusive human milk were included and infants exposed to fortifier but not formula were excluded (*n* = 180; Figure 1), following the statistical analysis pipeline outlined in Figure 2. The test confirmed that formula feeding increases the ARG load compared to an exclusively human milk diet. Formula feeding was associated with an approximately 70% increase in the relative abundance of ARGs in the meta-analysis (*n* = 180; gamma-distributed GLM, fold change for formula-containing diets compared with exclusively human milk–based diet: 1.69; 95% CI: 1.12–2.55; *P* = 0.013, adjusting for gestational age and 16S rRNA counts; **Figure 4**A). Formula was associated with an increase of 69% in the ARG load when not adjusting for other factors (*n* = 180; gamma-distributed GLM, fold change: 1.69; 95% CI: 1.11–2.57; *P* = 0.015). Gestational age and 16S rRNA counts, which were the other 2 explanatory variables in the model built using the train set, also significantly impacted the ARG load in the test set (*n* = 180; gamma-distributed GLM, fold change for gestational age/week of gestation: 0.94; 95% CI: 0.91–0.98; *P* = 0.0013; Figure 4A). The effect of 16S rRNA counts was small but significant (*P* < 0.01). We also normalized ARGs to the single copy *rpoB* gene and library size, but the results did not differ from those obtained using 16S rRNA normalized values (Supplemental Results; **Supplemental Table 1**).

Neither supplementation of the infant's own mother's milk with fortifier nor supplementation with donor milk had a significant effect on the ARG load (Figure 4B; *n =* 67; gamma-distributed GLM, *P* = 0.42 for fortifier and *P* = 0.91 for donor milk, adjusting for 16S rRNA counts and gestational age). The meta-analysis cohorts included 1 other study with infants fed fortified mother's milk and donor milk–fed infants (3). Again, these results support the idea that fortifier and donor milk supplementation have less impact than formula feeding on the neonatal gut ARG load. However, the analyses lack the statistical power necessary to draw definitive conclusions (power <0.5 for fortifier and power <0.1 for donor milk).

The median ARG load was higher in the formula-fed infants in all cohorts (Figure 4C), suggesting that formula-fed neonates may exhibit higher intestinal ARG loads, independent of the study design or gestational age. The most prevalent ARG classes differed between the 2 diets, especially for aminoglycoside and macrolide-lincosamide-streptogramin B (MLSB) ARGs, which were more abundant in infants fed formula (gamma-distributed GLM; *P* < 0.05; Figure 4D). Actinobacteria and Bacteroidia were significantly enriched in human milk–fed infants and Gammaproteobacteria was enriched in formula-fed infants (quasibinomial GLM, *P* < 0.05, Figure 4D).

The ARGs of formula-fed infants and infants fed exclusively human milk clustered separately (all cohorts, *n* = 206, including 26 infants not fed fortifier in the trainset; PERMANOVA, Horn-Morisita similarity: *R^2^* = 0.011; adjusted *P =* 0.004). Notably, several ARGs were enriched in formula-fed infants (*n* = 206; DESeq2: *P* < 0.05; Figure 4E). The enriched genes included potential ESBLs of the SHV type (*P* < 0.05; Figure 4E), the *mecA* gene encoding methicillin resistance in *Staphylococcus* species, and the MLSB resistance gene *ermC* encoding erythromycin resistance, typically in *Staphylococcus aureus*. The SHV, *mecA*, and *ermC* genes confer resistance phenotypes, all of which are highly relevant in NICUs (45). Sulphonamide resistance genes of the *sul1* type were more abundant in formula-fed infants, indicating the enrichment of class 1 integrons, which contribute to multidrug resistance in bacteria, including *Enterobacteriaceae* (46).

### De novo refinement of the initial model

Because our original cohort was small (*n* = 46), we reperformed de novo model selection for the gamma-distributed GLMs, modeling ARG loads on all collected variables in both the meta-analysis data set and trainset (*n* = 206; Supplemental Data 3). Infant and maternal antibiotic use, gestational age, infant age, delivery mode, 16S rRNA gene count, and diet had importance values >0 in our RF analysis, and we included them in the full de novo model. We also added sex, preeclampsia, history of NEC, and the type of antibiotics used, as they might also affect the ARG load. The results of the full de novo model are shown in Supplemental Data 4. The final refined de novo model included formula use, as expected, as well as gestational age, 16S rRNA counts, infant's age, and vancomycin and fortifier use. Formula-fed infants had 57% higher ARG loads (gamma-distributed GLM; *n* = 206; fold change 1.57; 95% CI: 1.12–2.19; *P* < 0.009; Supplemental Data 4). The model built using the preterm infant cohort explained approximately 43% of the ARG load variation, and the de novo model explained 74% of the variance (0.77 pseudo-*R*^2^ calculated using the Cragg-Uhler method).

Consistent with our data set for preterm infants, antibiotic use, which was treated as a binary categorical variable (yes or no), did not improve the model in the meta-analysis data set for neonates (*n* = 206; chi-squared test: *P* = 0.96; gamma-distributed GLM, fold change = 0.98; 95% CI: 0.47–2.03; *P* = 0.96; Supplemental Data 4). However, our study was not designed to investigate the effect of antibiotics specifically, and thus, it should not be concluded that antibiotics do not influence the ARG load. The result merely indicates that for our data set and model, the inclusion of antibiotics did not improve the model fit. Additional results of the possible effect of antibiotics are described in the Supplemental Results. Only 1 of the antibiotics given to the infants, vancomycin, was correlated significantly with the ARG load and was associated with a decreased ARG load in neonates (*n* = 206; gamma-distributed GLM: fold change 0.53; 95% CI: 0.30–0.92; *P* = 0.025). Vancomycin is often prescribed to treat infections caused by methicillin-resistant *Staphylococcus aureus* and *Staphylococcus epidermidis*, which are often multiresistant.

### Formula-fed infants have more potential pathogens possessing ARGs

**FIGURE 5:**
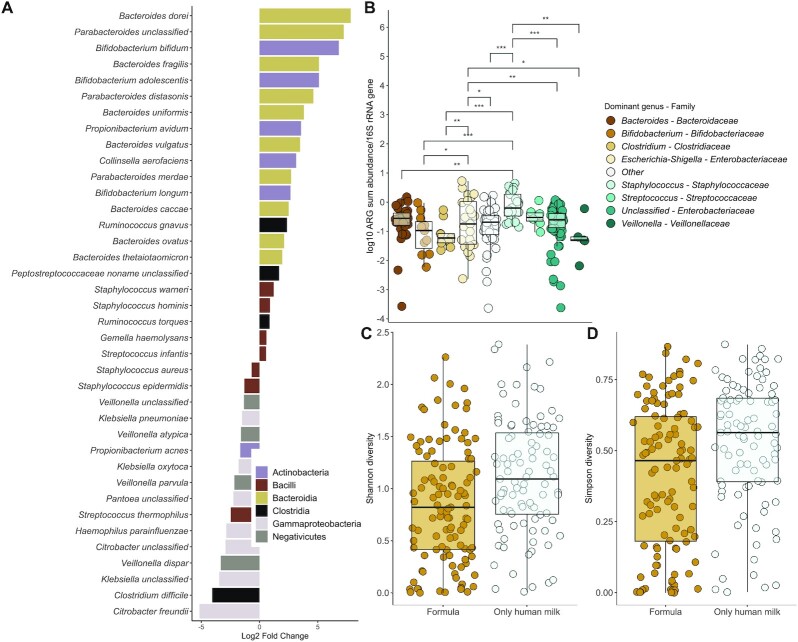
Microbial community changes linked to ARG abundances in the meta-analysis cohorts. (A) Species enriched or depleted in formula-fed neonates. Negative/positive values reflect significant enrichment/depletion in formula-fed neonates using DESeq2 analysis. Species with negative values are enriched in formula-fed infants, and species with positive values are depleted in infants fed formula, with an adjusted *P* value cutoff of 0.05 for reported species. (B) Relative ARG abundances in neonates whose guts are dominated by different genera. Significance levels are denoted as follows: *** = 0–0.001; ** = 0.001–0.01; * = 0.01–0.05. *Staphylococcus*-dominant infants had the highest relative abundances of ARGs (gamma distributed GLM: *n* = 242, adjusted *P* < 0.05; Supplemental Data 6). (C) Shannon and (D) Simpson (1—*D*) diversity indexes by formula consumption in neonates. Infants fed formula had significantly lower microbial community diversity than infants exclusively fed human milk gamma-distributed GLMs [*n* = 242; Shannon: 0.81 (95% CI: 0.68–0.96); Simpson: fold change, 0.81 (95% CI: 0.69–0.96); *P* = 0.01]. (B–D) The boxplot hinges represent 25% and 75% quantiles, and the centerline shows the median. Notches are calculated with the formula median ± 1.58 × IQR/sqrt (*n*). Abbreviations: ARG, antibiotic resistance gene; GLM, generalized linear mode.

There were significant differences between the intestinal microbial communities of infants fed any formula and infants exclusively fed human milk (*n* = 206). Genera belonging to the obligate anaerobic families *Bifidobacteriaceae, Veillonellaceae, Clostridiaceae, Lachnospiraceae*, and *Porphyromonadaceae* were depleted in formula-fed infants, and, in contrast, the facultative anaerobic families *Enterobacteriaceae, Staphyloccoccaceae*, and *Enterococcaceae* were enriched in formula-receiving subjects (Figure 4F; DESeq2: *P* < 0.05). In addition to genera, several potentially pathogenic species, including the facultative anaerobes *Staphylococcus aureus, Staphylococcus epidermidis, Klebsiella pneumoniae, Klebsiella oxytoca*, and the strict anaerobe *Clostridium difficile* (currently *Clostridioides difficile*) were enriched in formula-fed infants (DESeq2: *P* < 0.001; **Figure 5**A; Supplemental Data 5). In contrast, typical infant-associated species, such as *Bifidobacterium longum* and *Bacteroides* and *Parabacteroides* spp., were depleted in the formula-fed infants (*P* < 0.001; Figure 5A; Supplemental Data 5).

We observed that the infants who had microbial communities dominated by different genera (not accounting for differences in diet) had significantly different ARG loads. *Staphylococcus*-dominant infants had the highest relative abundances of ARGs (gamma-distributed GLM: *n* = 242; adjusted *P* < 0.05; Figure 5B; **Supplemental Data 6**). The ARGs also clustered distinctly by the dominant genus, confirming that the microbial community composition likely drives differences in the resistome as well (PERMANOVA: *n* = 242; adjusted *P* < 0.05; Supplemental Data 6; Supplemental Results; Figure 2). Somewhat to our surprise, infants fed formula had significantly lower microbial community diversity than infants exclusively fed human milk [gamma-distributed GLMs, *n* = 242; Shannon: 0.81 (95% CI: 0.68–0.96); Simpson, fold change: 0.81 (95% CI: 0.69–0.96); *P* = 0.01; Figure 5C and 5D; Supplemental Data 5). The opposite has been observed in older infants (16, 47, 48). However, our result is similar to previous observations in preterm infants sampled in the neonatal period (43).

## Discussion

Our study aimed to determine whether formula feeding in the neonatal period and early infancy affects the infant gut microbiota and ARG load. The primary outcome of the study was that formula feeding is associated with a 70% increase in ARG abundance in neonates compared to infants fed only human milk. Our finding was novel and was validated using several independent cohorts, suggesting that the effect of formula is generalizable in the neonatal population. The ARG load in the premature infant gut was nearly doubled in infants receiving formula compared to infants receiving only human milk.

In addition, the secondary outcome variables of gestational age and infant age affected the ARG load, with younger and more premature infants having higher ARG loads. Antibiotic exposure did not improve our model for ARG loads in full-term or preterm infants, even though antibiotic use as a whole increases the global prevalence of antibiotic-resistant genes (49). However, our study was not designed to investigate the role of antibiotics; antibiotic use was treated as a binary variable, many of the infants received antibiotics only for a few days, and often the sampling was done several days after the treatment had finished. The lack of a significant effect of antibiotic use on increases in the ARG load might also be partly due to antibiotics sometimes being used to treat infections caused by multiresistant pathogens carrying multiple resistance genes. Thus, treatment subsequently might decrease the ARG load when the abundance of the multiresistant pathogen is reduced, and surviving strains carry fewer resistance genes. We hypothesized this to be the case with vancomycin treatment of neonates, which was associated with a reduced ARG load. Overall, further studies are required to understand the relationships between premature birth, infant age, diet, antibiotic use, and ARG load.

Formula-fed neonates harbored increased abundances of *Enterobacteriaceae* and clinically relevant ARGs that can potentially confer methicillin resistance in *Staphylococcus aureus* and ESBL phenotypes compared to subjects fed human milk exclusively. Interestingly, *Enterobacteriaceae* have been suspected of contributing to NEC's pathogenesis, and prematurity and exposure to formula are well-established risk factors for NEC (50–53). Somewhat surprisingly, formula feeding reduced neonatal gut microbial diversity. Typically, older formula-fed infants have lower diversity than breastfed infants (16, 47, 48, 54). Exclusively breastfed infants often harbor high numbers of bifidobacteria, resulting in low diversity. However, bifidobacterial dominance may take longer to establish than domination by facultative anaerobes promoted by formula feeding, as the abundance of bifidobacteria is highest at 3–6 months of age (5, 55). Our results suggest that the changes in formula-fed infants’ intestinal environment may result in simple microbial communities enriched with ARG-carrying facultative anaerobes, in contrast to the more diverse and less antibiotic-resistant communities of infants only fed human milk. The current study showed that the ARG load and resistome relate to the microbial community structure, and taxa enriched in formula-fed infants correlate with a higher ARG abundance.

We did not include follow-up on the subjects to determine whether those infants who were fed formula or had higher ARG abundances had more infections caused by antibiotic-resistant bacteria or whether their health was impacted otherwise. We are limited to extrapolating from ARGs identified from shotgun metagenome data, which means that we cannot confirm that the ARGs are functional and confer resistance or identify the host of the ARG accurately. The identification of the ARGs is also reliant on short reads, which limits the resolution of distinguishing between variants. The findings of our study can be applied to practice in the clinic when it is known how ARG carriage impacts infant health. The effect of fortifiers on the ARG load should also be more extensively investigated to ensure that their use is beneficial compared to infant formula.

In conclusion, our data suggest that a diet containing only human milk in the first months of life reduces the ARG load by modulating the microbial community to favor non-ARG-carrying bacteria. The results add to the body of knowledge on breastfeeding's health benefits in both full-term and preterm infants. Infants born prematurely are at particular risk of acquiring severe and life-threatening infections. Thus, increased ARG loads in formula-fed infants and the enrichment of potentially pathogenic bacteria are concerning. Supplementing human milk with fortifier was not associated with high ARG abundances, which is reassuring in light of current nutrition guidelines for infants born prematurely. Clinicians are encouraged to be prudent when using antibiotics to limit the spread of antibiotic resistance. Accordingly, our results suggest that clinicians should assess the risks associated with elevated antibiotic resistance gene abundance coupled with increased opportunistic pathogen prevalence in the infant gut microbiota when making choices about feeding methods, since formula feeding was associated with increases in both. Our results suggest that when necessary to provide additional nutritional support, supplementation of human milk with fortifier might have a smaller impact on the intestinal ARG load than transitioning to infant formula, potentially reducing the risk for infections caused by antibiotic-resistant bacteria.

## Supplementary Material

nqab353_Supplemental_Files

## Data Availability

The sequence data are available from the National Center for Biotechnology Information and Sequence Read Archive under the accession number PRJNA532310. Data used for the meta-analysis are available from https://www.ncbi.nlm.nih.gov/sra, and the accession numbers for sequences are listed in the Supplemental Materials of this study. All custom codes are available from https://github.com/KatariinaParnanen; analytic code will be made publicly and freely available without restriction at https://github.com/KatariinaParnanen.

## Notes

This research was supported by the Academy of Finland. KMMP received funding from the MBDP (Microbiology and Biotechnology Doctoral Programme, University of Helsinki). doctoral program at the University of Helsinki, Finland. SLK received funding from The Gerber Foundation. RS is funded by the Sigrid Juselius Foundation, Finland.

The funding agencies had no role in the design, analysis, or writing of this article.

Supplemental Results, Supplemental Notes 1 and 2, Supplemental Data 1–6, and Supplemental Table 1 are available from the “Supplementary data” link in the online posting of the article and from the same link in the online table of contents at https://academic.oup.com/ajcn/.

Abbreviations used: AIC, Akaike information criterion; ARB, antibiotic-resistant bacteria; ARG, antibiotic resistance gene; ESBL, extended spectrum beta-lactamase; GLM, generalized linear mode; MGE, mobile genetic element; MLSB, macrolide-lincosamide-streptogramin B; NEC, necrotizing enterocolitis; NICU, neonatal intensive care unit; PERMANOVA, permutational multivariate analysis of variance; PSHMC, Penn State Hershey Medical Center; RF, random forest; rRNA, ribosomal RNA.
