# Supplementary material for: Early-life formula feeding is associated with infant gut microbiota alterations and an increased antibiotic resistance load
Source: Am J Clin Nutr. 2021 Oct 22;115(2):407–21. doi: 10.1093/ajcn/nqab353 (PMC8827105; doi:10.1093/ajcn/nqab353)
Supplement: nqab353_Supplemental_Files [file nqab353_supplemental_files.zip › Supplementary material.docx]

**Early-life formula feeding is associated with infant gut microbiota alterations and an increased antibiotic resistance load, Pärnänen et al.,**

**Online Supplementary Material**

**This file includes:**

Supplementary Results

Supplementary Figure 1

Supplementary Figure 2

Supplementary Table 1

Supplementary Note 1

Supplementary Note 2

Supplementary Text

**Supplementary Results**

## **Resistome is correlated with community composition**

The resistome and microbial community composition in our preterm infant cohort were significantly correlated (Mantel’s test for Horn-Morisita similarity index: *r* = 0.21, *p* = 0.001). Moreover, there was a significant correlation between MGEs and the microbial species (Mantel’s test for Horn-Morisita similarity index: *r* = 0.27, *p* = 0.001).

The microbiota formed four clusters with *Veillonella*, *Klebsiella*, *Escherichia*, or other (dominance by a genus which was dominant in only 1–2 infants) (Supplementary Figure 1A). The metabolic genes were consistent between the microbial community composition and the metabolic pathways (PERMANOVA: false discovery rate adjusted *p* < 0.01, Supplementary Figure 1B; Supplementary Data 3). ARGs clustered separately in *Escherichia-* and *Klebsiella-*dominated infants (PERMANOVA: false discovery rate adjusted *p* < 0.01; Supplementary Figure 1C; Supplementary Data 3). MGEs clustered separately in infants dominated by other and *Escherichia*, and *Klebsiella* and *Escherichia* (PERMANOVA: false discovery rate adjusted *p* < 0.05; Supplementary Figure and 1D; Supplementary Data 3).

The gut communities were often dominated by one genus, which in some cases reached > 90% relative abundance. Overall, the diversity of the microbial community in the preterm infant gut was very low with Shannon diversities ranging from 0.17 to 3.2, with a mean of 1.9. There were no significant differences in the Shannon diversities between infants dominated by different genera (analysis of variance, AOV: false discovery rate adjusted *p* > 0.05). Metabolic gene diversity was higher in *Veillonella-*dominated infants compared to other infants (AOV: false discovery rate adjusted *p* < 0.05).

# **Alternative normalizations using the single copy *rpoB* gene and library size**

The possibility of using a single copy marker gene was explored by using *rpoB* gene counts to normalize ARG data, since there has been questions whether 16S rRNA gene is appropriate for normalization when it is know that bacteria harbor more than one copy of the gene and some taxa have more copies than others. We explored this possibility by using HMMER3 and the *rpoB* Hidden Markow model (HMM) retrieved from Pfam database (33,34). After running the analysis with e^-02^ threshold on sequences translated to amino acids in all six reading frames, we did not identify an adequate number of matches from all of the metagenomic libraries to be able to use the *rpoB* HMM model for normalization purposes. We observed that there was a less than r^2^<0.4 correlation with *rpoB* and 16S rRNA gene counts as well as correlation to metagenome read counts. Some libraries produced less than ten hits to *rpoB* genes skewing the results. However, when those samples which had less than 200 *rpoB* gene copies were excluded the estimates of the GLM estimated remained similar for formula (*n* = 139, fold change 2.23, (95% CI 1.36, 3.7), *p* = 0.0018), gestational age (fold change 0.94, (95% CI 0.88, 0.99), *p* = 0.04), and age (fold change 0.95 (95% CI 0.91, 0.99)). As a result of the *rpoB* normalization experiments, we concluded that *rpoB* gene normalization is not currently feasible for infant gut metagenome data since in several samples far fewer *rpoB* gene copies were identified using HMM models than expected. Specifically, the samples which had a dominance of either *Bifidobacterium* of *Bacteroidetes* has less *rpoB* genes than expected (Supplementary Table 1). The *rpoB* gene is less conserved than the 16S rRNA gene which might explain why it is difficult to identify *rpoB* genes from short metagenomic sequencing reads. Another alternative explanation is that the Pfam database’s HMM model for *rpoB* is lacking in representative sequences from those genera.

The GLM with gestational age and age as explanatory variables together with formula was also run with completely unnormalized values, and there the estimate for fold change caused by formula on the ARG load was estimated to be 2.1 (*n =* 206, GLM with gamma distribution (95% CI 1.39, 3.18) , *p* = 0.005). Also, normalization to library size in millions of sequences was used and the increase in ARG load caused by formula was 50% ((*n =* 206, GLM with gamma distribution *p* = 0.016, 95% CI from 1.08 to 2.08).

## **Effect of antibiotics and other clinical characteristics on ARG load**

Consistent with our dataset for preterm infants, antibiotic use, which was treated as a binary categorical variable (yes or no), did not improve the model in the meta-analysis dataset for neonates (*n* = 206, chi-squared test: *p* = 0.96, and gamma distributed GLM: fold change = 0.98, 95% CI from 0.47 to 2.03, *p* = 0.96; Supplementary Data 5). However, gestational age and age were correlated with antibiotic use (Supplementary Data 5), which confounded our ability to fully differentiate between their effects on the ARG load. Nevertheless, age and gestational age were also significantly negatively correlated with ARG abundance in a subset of infants who were treated with antibiotics (gamma distributed GLM: *p* < for both 0.01, Supplementary Data 5), confirming that the effects of gestational age and age are not caused by their correlation with antibiotic use.

The effect of being fed any formula on the ARG load did not reach statistical significance in neonates who were not treated with antibiotics (*n* = 102, gamma distributed GLM: *p* > 0.05, Supplementary Data 5), although the analysis lacked statistical power (power estimate < 0.5). Nevertheless, we could detect an effect of being fed formula during the first month of life on ARG load later in infancy in subjects who were not treated with antibiotics in the Shao et al. cohort(17) (gamma distributed GLM controlling for age, *n* = 269, *p* = 0.0006, fold change 1.39, CI from 1.15 to 1.67).

A model with an interaction term between antibiotics and formula was fitted for ARG load during the first month of life, and the interaction was significant (n = 206, gamma distributed GLM: fold change 2.8 for infants with antibiotic treatment and formula compared to infants without formula and treatment, (95% CI 1.4, 5.6), *p* = 0.0041), which might suggest that antibiotic treatment increases susceptibility to colonization by ARG-carrying bacteria during the first month of life if an infant is formula-fed. However, it is not possible to infer whether this potential interaction observed in neonates is related to differences in gestational age in antibiotic treated versus non-treated infants (mean gestational age 29 weeks versus 38 weeks), since most preterm infants are routinely treated with antibiotics.

Antibiotic treatment did not affect species diversity in the meta-analysis dataset (n = 206, fold change ≈ 1.0, *p =* 0.91 – 0.96, Supplementary Data 6). Antibiotic treatment with specific antibiotics has been previously linked to decreased diversity in preterm infants(3,4). Not differentiating between antibiotic classes can mask the effects that are caused by specific antibiotic classes and therefore this result should be interpreted with caution.

Delivery mode, maternal and infant diagnoses, and maternal and infant antibiotic use did not have significant effects on the ARG load of the infants. Sex was weakly significant in the Bäckhed dataset (5), but was not included in the final model as the association was only found in that dataset. Infant NEC and maternal pre-eclampsia status were available for all the infants included in the meta-analysis, but the effects of other infant and maternal diagnoses were investigated within individual studies.

## **Formula feeding has a long-term impact on intestinal ARG load**

To study the effect of formula on the total intestinal ARG load in full-term infants, we investigated gut ARG loads in a large metagenomic cohort of full-term neonates and the full-term infants from Bäckhed et al. (5,17). Formula feeding did not significantly explain ARG load in these full-term infants sampled in the first week of life (*n* = 315, see Supplementary Note 1 for list of ENA accession numbers, Supplementary Figure 2A, *p* > 0.05).

Since we did not observe the effect of formula in the first week of life in full-term infants, we investigated if formula feeding before introducing solid foods increases the ARG load later in infancy. We included subjects from the Bäckhed et al. and Shao et al. studies sampled at ~12 and ~8 months of age (total *n* = 315; see Supplementary Note 2 for list of ENA accession numbers) (5,17). We used metadata on the mode of feeding (during either the first Shao et al. or first four Bäckhed et al. months of life) collected ~8 months before the sampling in later infancy to predict the ARG load. Formula feeding in the first months of life was associated with an increased ARG load later in infancy (Figure 2B, gamma-distributed GLM, *n* = 315, fold change = 1.40, (95% CI 1.18, 1.65), *p =* 0.001) despite an absence of this effect in the first week of life. Infant age negatively correlated with the ARG load and was included in the model (gamma distributed GLM, fold change per one month of age 0.96, (95% CI 0.92, 0.99), *p* = 0.01).

**Further investigation of possible confounders**

To further investigate the finding, we separately analysed each of the cohorts. All of the metadata available in Shao et al., and Bäckhed et all., cohorts was used. In Shao et al. we investigated factors such as the mode of current feeding (mixed versus mixed without any breastfeeding) and delivery mode, as well as antibiotics in hospital and after hospital, sex and maternal IAP, but these were not significant. The only significant effect was for the infant formula in the first month of life (gama distributed GLM, fold change 1.32, 95%CI 1.09, 1.60, *p* = 0.0043, *n* = 246). In Bäckhed et al.,we investigated the effect of current diet (any breastfeeding at age 12 months, all had solid food at the time of sampling), antibiotics in the first four months of life (times), times of antibiotic treatments until 12 Months of age.

In addition we investigated the impact of maternal antibiotic load in both of the datasets, to account for possible maternal effect, or environmental effects affecting ARG load related to the infants home environment shared with the mother. There were 52 mother infant pairs available in the Shao et al. and 69 mother-infant pairs in the Bäckhed et al. cohorts with mother, neonatal and the infancy sample available. Formula exposure in the first months of life was correlated with higher ARG load also for controlling for maternal ARG load (fold change 1.27, 95%CI 1.02 to 1.58, p= 0.036, controlling also for study and delivery mode). Maternal ARG load was correlated with infant ARG load (gamma distributed GLM, controlling for study, delivery mode and formula exposure in the first months of infancy fold change 2.00, 95%CI 1.21, 3.28, *p* = 0.01, *n* = 121).

**Microbiota changes observed in formula exposed full-term infants**

Full-term infants fed any formula in the first months of life also exhibited differences in several bacterial species’ abundances later in infancy. The most notable increase associated with formula feeding was in *Klebsiella* species’ abundances (Supplementary Figure 2C, DESeq2, false discovery rate adjusted *p*-value < 0.05). These prelimary results corroborate the finding that being fed formula during early life is correlated with higher ARG load through modifying the intestinal microbial community composition. Possibly having a long term effect, which changes persisting even after introducing solid foods. However, our results should be treated as preliminary since we did not have information for the age when solids were introduced and when breasfeeding was terminated.

We tested whether there was an effect of delivery mode on ARG load in the first week of life when the full-term neonates were sampled. We observed that cesarean section delivery was associated with an increased ARG load in the Shao et al. cohort, but not in the full-term neonates in the meta-analysis. Other clinical parameters (Supplementary Data 4) did not significantly affect ARG load.

We proceeded to investigate whether the antibiotic resistance load of the first week of life was associated with a higher resistance load later in infancy. Still, we found no correlations with ARG load in any of the cohorts (~4-month-old infants from Baumann-Dudenhoeffer et al. and Bäckhed et al., or later in infancy from Baumann-Dudenhoeffer et al., Bäckhed et al. and Shao et al.; gamma-distributed GLM, n = 348) (5,16,17). However, the ARG loads at ~4 months of age correlated with ARG loads at ~8 months of age (gamma distributed GLM controlling for formula feeding status at four months of age, *n* = 315, fold change 2.32, (95% CI 1.24, 4.35), *p* = 0.01,). The results suggest that antibiotic resistance genes stabilize sometime after the first week of life, and their levels persist for several months.


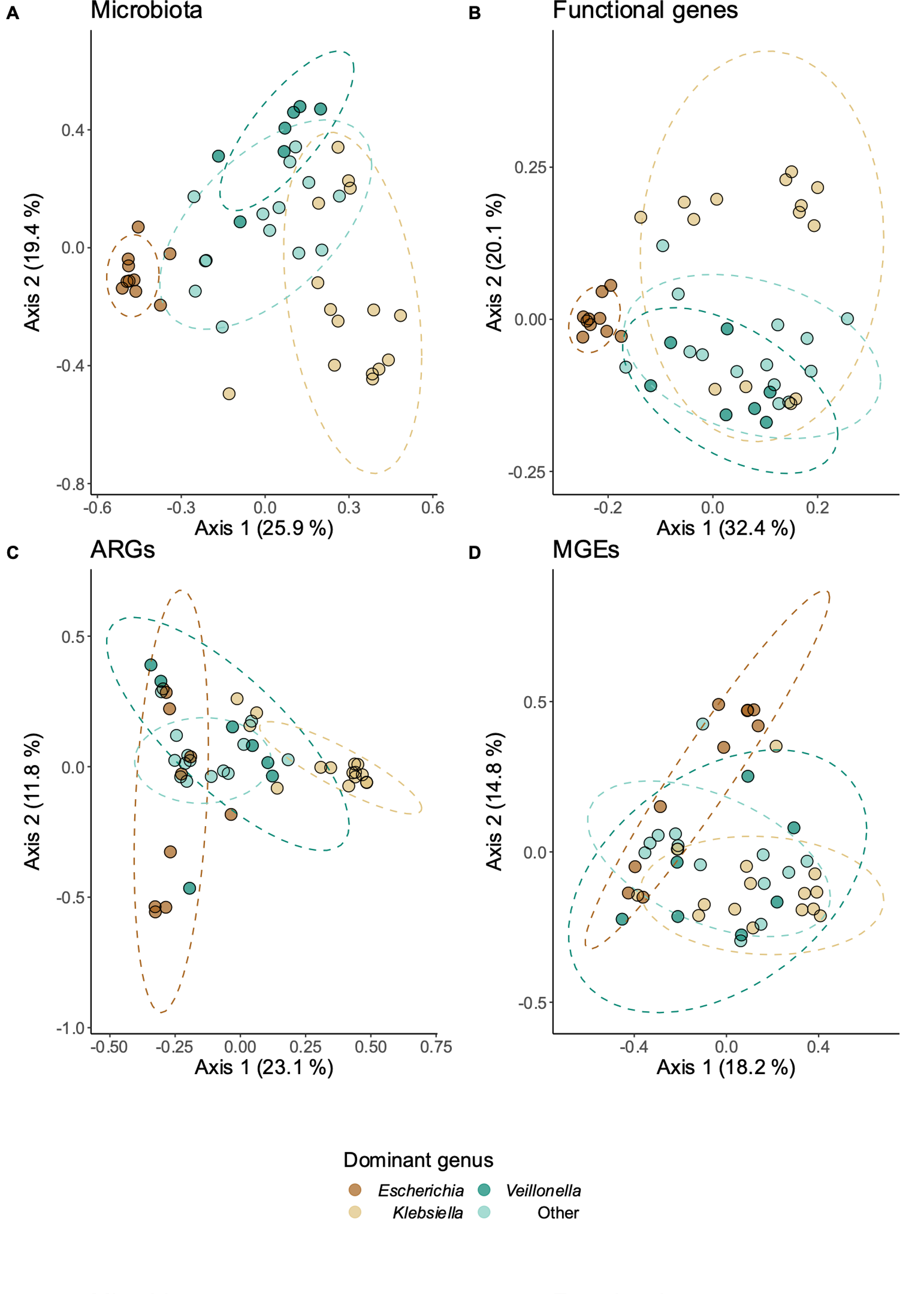


**Supplementary Figure 1. Clustering of microbial communities, functional gene pathways, ARGs and MGEs based on dominant species.** **(A)**, Ordination of microbial communities using MetaPhlAn2. **(B)**, Ordination of metabolic genes using enzyme categories. (**C)**, Ordination of ARGs. **(D)**, Ordination of MGEs. Ordinations have been performed using the Horn-Morisita similarity index with principal co-ordinate analysis (PCoA). Variation explained by axes 1 and 2 is denoted in percentages. Confidence ellipses are shown for the three most common dominant genera using 90% confidence levels. *Klebsiella* was the most common dominant genus (*n* = 15), followed by *Escherichia* (n = 10) and *Veillonella* (*n* = 7). The *n* for the analyses was 46.


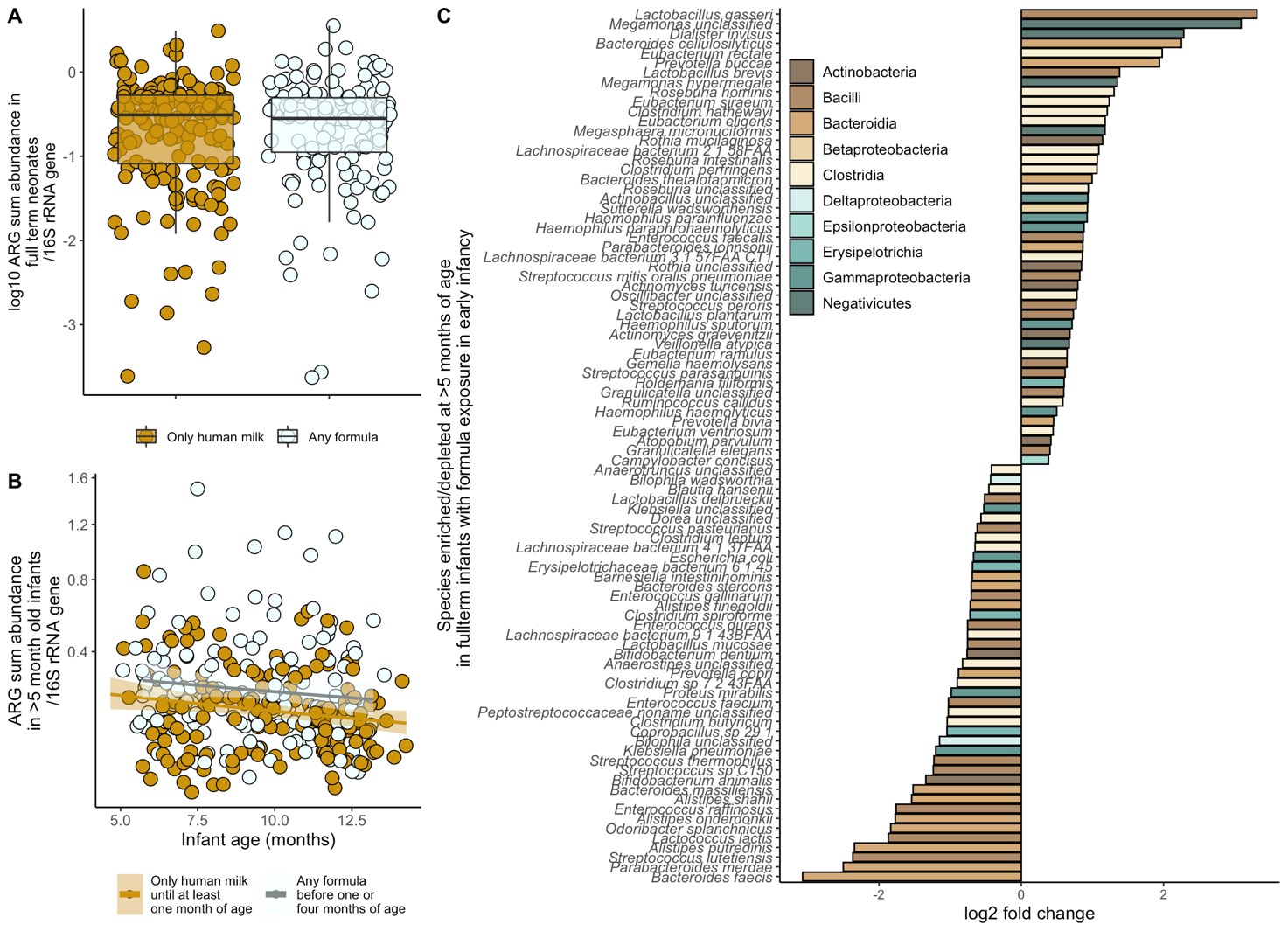


**Supplementary Figure 2. Effect of early life formula exposure on resistome and intestinal microbiome in full-term infants. (A)**, Differences in relative ARG sum abundance in full-term neonates related to formula feeding during the first week of life, *n* = 315. The boxplot hinges represent 25% and 75% percentiles, and the centerline the median. Notches are calculated with the formula median ± 1.58 × interquartile range/sqrt (*n*). (**B)**, Differences in relative ARG sum abundance related to age and formula exposure during the first (Bäckhed et al.) or first four months (Shao et al.) of life in full-term > 5-month-old infants, *n* = 315. Regression line is fitted using a gamma-distributed GLM. **(C)**, Species enriched or depleted during late infancy (six to 12 months of age) in full-term infants fed formula before 4-months of age and the introduction of solids, *n* = 315. Analysis was done using DESeq2. Negative values reflect significant enrichment in infants fed formula and positive values significant enrichment in infants only fed human milk (adjusted *p* < 0.05).

**Supplementary Table 1. Differences in *rpoB* gene counts for different dominant taxa.**

| **Linear hypotheses** | **Estimate** | **Fold change** | **Std. Error** | **z-value** | **Pr(>\|z\|)**  **(Adjusted)** |  |
| --- | --- | --- | --- | --- | --- | --- |
| Bifidobacterium-Bacteroides = 0 | 1.029932 | 2.80087537 | 0.545431 | 1.888 | 0.6969 |  |
| Clostridium-Bacteroides = 0 | 1.992242 | 7.33195359 | 0.523222 | 3.808 | <0.01 | ** |
| Enterobacter-Bacteroides = 0 | 2.400714 | 11.0310497 | 0.494897 | 4.851 | <0.01 | *** |
| Enterococcus-Bacteroides = 0 | 2.028873 | 7.60551012 | 0.51069 | 3.973 | <0.01 | ** |
| Escherichia-Bacteroides = 0 | 2.188677 | 8.92339964 | 0.48335 | 4.528 | <0.01 | *** |
| Haemophilus-Bacteroides = 0 | 2.379566 | 10.8002146 | 0.499756 | 4.761 | <0.01 | *** |
| Klebsiella-Bacteroides = 0 | 2.508329 | 12.2843857 | 0.481554 | 5.209 | <0.01 | *** |
| Staphylococcus-Bacteroides = 0 | 2.279678 | 9.77353283 | 0.495321 | 4.602 | <0.01 | *** |
| Veillonella-Bacteroides = 0 | 2.000107 | 7.38984677 | 0.52296 | 3.825 | <0.01 | ** |
| Other-Bacteroides = 0 | 1.581548 | 4.8624771 | 0.491387 | 3.219 | 0.043 | * |
| Clostridium-Bifidobacterium = 0 | 0.96231 | 2.61773647 | 0.357253 | 2.694 | 0.1806 |  |
| Enterobacter-Bifidobacterium = 0 | 1.370782 | 3.93842934 | 0.314311 | 4.361 | <0.01 | *** |
| Enterococcus-Bifidobacterium = 0 | 0.998941 | 2.71540469 | 0.338634 | 2.95 | 0.0943 | . |
| Escherichia-Bifidobacterium = 0 | 1.158745 | 3.18593242 | 0.295798 | 3.917 | <0.01 | ** |
| Haemophilus-Bifidobacterium = 0 | 1.349634 | 3.85601397 | 0.321908 | 4.193 | <0.01 | ** |
| Klebsiella-Bifidobacterium = 0 | 1.478397 | 4.38590943 | 0.292853 | 5.048 | <0.01 | *** |
| Staphylococcus-Bifidobacterium = 0 | 1.249746 | 3.48945652 | 0.31498 | 3.968 | <0.01 | ** |
| Veillonella-Bifidobacterium = 0 | 0.970175 | 2.63840614 | 0.35687 | 2.719 | 0.1703 |  |
| Other-Bifidobacterium = 0 | 0.551615 | 1.73605448 | 0.308756 | 1.787 | 0.7639 |  |
| Enterobacter-Clostridium = 0 | 0.408472 | 1.50451713 | 0.273965 | 1.491 | 0.9112 |  |
| Enterococcus-Clostridium = 0 | 0.036631 | 1.03731018 | 0.30156 | 0.121 | 1 |  |
| Escherichia-Clostridium = 0 | 0.196435 | 1.21705621 | 0.252511 | 0.778 | 0.9994 |  |
| Haemophilus-Clostridium = 0 | 0.387324 | 1.47303368 | 0.282649 | 1.37 | 0.9478 |  |
| Klebsiella-Clostridium = 0 | 0.516087 | 1.67545874 | 0.249055 | 2.072 | 0.5642 |  |
| Staphylococcus-Clostridium = 0 | 0.287436 | 1.33300528 | 0.274732 | 1.046 | 0.9927 |  |
| Veillonella-Clostridium = 0 | 0.007865 | 1.00789601 | 0.321903 | 0.024 | 1 |  |
| Other-Clostridium = 0 | -0.410694 | 0.66318984 | 0.267574 | -1.535 | 0.8944 |  |
| Enterococcus-Enterobacter = 0 | -0.371841 | 0.68946386 | 0.2492 | -1.492 | 0.9107 |  |
| Escherichia-Enterobacter = 0 | -0.212038 | 0.80893396 | 0.186878 | -1.135 | 0.9863 |  |
| Haemophilus-Enterobacter = 0 | -0.021148 | 0.97907405 | 0.225947 | -0.094 | 1 |  |
| Klebsiella-Enterobacter = 0 | 0.107614 | 1.11361781 | 0.182182 | 0.591 | 0.9999 |  |
| Staphylococcus-Enterobacter = 0 | -0.121037 | 0.88600118 | 0.215961 | -0.56 | 1 |  |
| Veillonella-Enterobacter = 0 | -0.400607 | 0.66991329 | 0.273465 | -1.465 | 0.9202 |  |
| Other-Enterobacter = 0 | -0.819167 | 0.44079869 | 0.206779 | -3.962 | <0.01 | ** |
| Escherichia-Enterococcus = 0 | 0.159804 | 1.17328089 | 0.2254 | 0.709 | 0.9997 |  |
| Haemophilus-Enterococcus = 0 | 0.350693 | 1.4200513 | 0.258715 | 1.356 | 0.9512 |  |
| Klebsiella-Enterococcus = 0 | 0.479456 | 1.6151955 | 0.221522 | 2.164 | 0.4968 |  |
| Staphylococcus-Enterococcus = 0 | 0.250805 | 1.28505947 | 0.250042 | 1.003 | 0.9947 |  |
| Veillonella-Enterococcus = 0 | -0.028766 | 0.9716438 | 0.301106 | -0.096 | 1 |  |
| Other-Enterococcus = 0 | -0.447325 | 0.63933609 | 0.242155 | -1.847 | 0.7249 |  |
| Haemophilus-Escherichia = 0 | 0.19089 | 1.21032631 | 0.199391 | 0.957 | 0.9964 |  |
| Klebsiella-Escherichia = 0 | 0.319652 | 1.37664861 | 0.14797 | 2.16 | 0.5001 |  |
| Staphylococcus-Escherichia = 0 | 0.091001 | 1.0952701 | 0.188 | 0.484 | 1 |  |
| Veillonella-Escherichia = 0 | -0.188569 | 0.82814336 | 0.251968 | -0.748 | 0.9996 |  |
| Other-Escherichia = 0 | -0.607129 | 0.54491307 | 0.177376 | -3.423 | 0.0228 | * |
| Klebsiella-Haemophilus = 0 | 0.128762 | 1.13741939 | 0.194996 | 0.66 | 0.9999 |  |
| Staphylococcus-Haemophilus = 0 | -0.099889 | 0.90493786 | 0.226876 | -0.44 | 1 |  |
| Veillonella-Haemophilus = 0 | -0.379459 | 0.68423148 | 0.282164 | -1.345 | 0.9539 |  |
| Other-Haemophilus = 0 | -0.798019 | 0.45021997 | 0.218153 | -3.658 | <0.01 | ** |
| Staphylococcus-Klebsiella = 0 | -0.228651 | 0.79560615 | 0.183333 | -1.247 | 0.9725 |  |
| Veillonella-Klebsiella = 0 | -0.508221 | 0.60156481 | 0.248505 | -2.045 | 0.5844 |  |
| Other-Klebsiella = 0 | -0.926781 | 0.39582583 | 0.172421 | -5.375 | <0.01 | *** |
| Veillonella-Staphylococcus = 0 | -0.27957 | 0.7561088 | 0.274233 | -1.019 | 0.994 |  |
| Other-Staphylococcus = 0 | -0.69813 | 0.49751479 | 0.207794 | -3.36 | 0.0275 | * |
| Other-Veillonella = 0 | -0.41856 | 0.65799365 | 0.267062 | -1.567 | 0.8807 |  |

Modeling is done using GLMs with quasipoisson distribution and 16S rRNA counts as offset in the model and Tukey’s *post hoc* test results are shown. The p-values are adjusted for multiple comparisons.

**Supplementary Note 1.** List of ENA accession numbers of the Shao et al. study for samples used in the analysis of formula impact on ARG load in full-term infants

|  |
| --- |
| ERS3420791 |
| ERS3420792 |
| ERS3420795 |
| ERS3420804 |
| ERS3420805 |
| ERS3420807 |
| ERS3420809 |
| ERS3420810 |
| ERS3420812 |
| ERS3420817 |
| ERS3420820 |
| ERS3420822 |
| ERS3420825 |
| ERS3420827 |
| ERS3420828 |
| ERS3420830 |
| ERS3420832 |
| ERS3420835 |
| ERS3420836 |
| ERS3420837 |
| ERS3420838 |
| ERS3420842 |
| ERS3420844 |
| ERS3420845 |
| ERS3420847 |
| ERS3420850 |
| ERS3420854 |
| ERS3420857 |
| ERS3420860 |
| ERS3420861 |
| ERS3420864 |
| ERS3420866 |
| ERS3420868 |
| ERS3420871 |
| ERS3420873 |
| ERS3420877 |
| ERS3420881 |
| ERS3420887 |
| ERS3420889 |
| ERS3420891 |
| ERS3420895 |
| ERS3420898 |
| ERS3420902 |
| ERS3420905 |
| ERS3420908 |
| ERS3420910 |
| ERS3420913 |
| ERS3420918 |
| ERS3420921 |
| ERS3420924 |
| ERS3420926 |
| ERS3420929 |
| ERS3420935 |
| ERS3420940 |
| ERS3420942 |
| ERS3420946 |
| ERS3420949 |
| ERS3420953 |
| ERS3420957 |
| ERS3420960 |
| ERS3420963 |
| ERS3420965 |
| ERS3420967 |
| ERS3420971 |
| ERS3420976 |
| ERS3420979 |
| ERS3420986 |
| ERS3420990 |
| ERS3420993 |
| ERS3420996 |
| ERS3421000 |
| ERS3421003 |
| ERS3421007 |
| ERS3421009 |
| ERS3421019 |
| ERS3421026 |
| ERS3421029 |
| ERS3421034 |
| ERS3421036 |
| ERS3421039 |
| ERS3421043 |
| ERS3421047 |
| ERS3421051 |
| ERS3421054 |
| ERS3421059 |
| ERS3421064 |
| ERS3421070 |
| ERS3421072 |
| ERS3421075 |
| ERS3421077 |
| ERS3421081 |
| ERS3421088 |
| ERS3421094 |
| ERS3421096 |
| ERS3421100 |
| ERS3421105 |
| ERS3421108 |
| ERS3421111 |
| ERS3421115 |
| ERS3421118 |
| ERS3421122 |
| ERS3421126 |
| ERS3421131 |
| ERS3421135 |
| ERS3421142 |
| ERS3421146 |
| ERS3421150 |
| ERS3421151 |
| ERS3421153 |
| ERS3421156 |
| ERS3421157 |
| ERS3421159 |
| ERS3421163 |
| ERS3421166 |
| ERS3421168 |
| ERS3421170 |
| ERS3421173 |
| ERS3421176 |
| ERS3421181 |
| ERS3421183 |
| ERS3421184 |
| ERS3421188 |
| ERS3421607 |
| ERS3421610 |
| ERS3421612 |
| ERS3421614 |
| ERS3421617 |
| ERS3421619 |
| ERS3421623 |
| ERS3421625 |
| ERS3421628 |
| ERS3421630 |
| ERS3421633 |
| ERS3421635 |
| ERS3421638 |
| ERS3421641 |
| ERS3421645 |
| ERS3421648 |
| ERS3421650 |
| ERS3421653 |
| ERS3421655 |
| ERS3421657 |
| ERS3421664 |
| ERS3421665 |
| ERS3421669 |
| ERS3421671 |
| ERS3421673 |
| ERS3421676 |
| ERS3421680 |
| ERS3421682 |
| ERS3421685 |
| ERS3421687 |
| ERS3421689 |
| ERS3421693 |
| ERS3421697 |
| ERS3421699 |
| ERS3421702 |
| ERS3421707 |
| ERS3421715 |
| ERS3421720 |
| ERS3421723 |
| ERS3421725 |
| ERS3421728 |
| ERS3421729 |
| ERS3421732 |
| ERS3421733 |
| ERS3421736 |
| ERS3421739 |
| ERS3421742 |
| ERS3421744 |
| ERS3421747 |
| ERS3421752 |
| ERS3421753 |
| ERS3421758 |
| ERS3421759 |
| ERS3421761 |
| ERS3421766 |
| ERS3421768 |
| ERS3421771 |
| ERS3421773 |
| ERS3421779 |
| ERS3421781 |
| ERS3421785 |
| ERS3421787 |
| ERS3421789 |
| ERS3421792 |
| ERS3421795 |
| ERS3421797 |
| ERS3421798 |
| ERS3421802 |
| ERS3421804 |
| ERS3421807 |
| ERS3421813 |
| ERS3421818 |
| ERS3421826 |
| ERS3421829 |
| ERS3421833 |
| ERS3421835 |
| ERS3421838 |
| ERS3421841 |
| ERS3421844 |
| ERS3421849 |
| ERS3421853 |
| ERS3421857 |
| ERS3421861 |
| ERS3421865 |
| ERS3421869 |
| ERS3421873 |
| ERS3421878 |
| ERS3421881 |
| ERS3421887 |
| ERS3421892 |
| ERS3421894 |
| ERS3421897 |
| ERS3421902 |
| ERS3421905 |
| ERS3421911 |
| ERS3421912 |
| ERS3421915 |
| ERS3421917 |
| ERS3421923 |
| ERS3421927 |
| ERS3421929 |
| ERS3421932 |
| ERS3421939 |
| ERS3421943 |
| ERS3421946 |
| ERS3421949 |
| ERS3421951 |
| ERS3421954 |
| ERS3421958 |
| ERS3421961 |
| ERS3421968 |
| ERS3421971 |
| ERS3421974 |
| ERS3421977 |
| ERS3421978 |
| ERS3421982 |
| ERS3421985 |
| ERS3421994 |
| ERS3421996 |
| ERS3421998 |
| ERS3422002 |
| ERS3422007 |
| ERS3422011 |
| ERS3422016 |
| ERS3422019 |
| ERS3422022 |
| ERS3422025 |
| ERS3422032 |
| ERS3422035 |
| ERS3422039 |
| ERS3422047 |
| ERS3422048 |
| ERS3422052 |
| ERS3422055 |
| ERS3422059 |
| ERS3422062 |
| ERS3422063 |
| ERS3422067 |
| ERS3422070 |
| ERS3422075 |
| ERS3422098 |
| ERS3422102 |
| ERS3422107 |
| ERS3422110 |
| ERS3422112 |
| ERS3422116 |
| ERS3422118 |
| ERS3422119 |
| ERS3422122 |
| ERS3422125 |
| ERS3422129 |
| ERS3422132 |
| ERS3422135 |
| ERS3422139 |
| ERS3422142 |
| ERS3422145 |
| ERS3422156 |
| ERS3422158 |
| ERS3422163 |
| ERS3422168 |
| ERS3422171 |
| ERS3422175 |
| ERS3422177 |
| ERS3422179 |
| ERS3422185 |
| ERS3422187 |
| ERS3422191 |
| ERS3422201 |
| ERS3422208 |
| ERS3422212 |
| ERS3422218 |
| ERS3422221 |
| ERS3422223 |
| ERS3422226 |
| ERS3422231 |
| ERS3422234 |
| ERS3422238 |
| ERS3422241 |
| ERS3422243 |
| ERS3422247 |
| ERS3422249 |
| ERS3422252 |
| ERS3422260 |
| ERS3422263 |
| ERS3422264 |
| ERS3422267 |
| ERS3422268 |
| ERS3422270 |
| ERS3422272 |
| ERS3422274 |
| ERS3422276 |
| ERS3422280 |
| ERS3422281 |
| ERS3422283 |
| ERS3422287 |
| ERS3422294 |
| ERS3422299 |
| ERS3422301 |
| ERS3422303 |
| ERS3422306 |
| ERS3422308 |
| ERS3422312 |
| ERS3422313 |
| ERS3422315 |
| ERS3422318 |
| ERS3422320 |
| ERS3422321 |
| ERS3422323 |
| ERS3422326 |
| ERS3422327 |
| ERS3422331 |
| ERS3422332 |
| ERS3422338 |
| ERS3422340 |
| ERS3422342 |
| ERS3422344 |
| ERS3422348 |
| ERS3422350 |
| ERS3422352 |
| ERS3422354 |
| ERS3422356 |
| ERS3422358 |
| ERS3422360 |
| ERS3422363 |
| ERS3422364 |
| ERS3422366 |
| ERS3422368 |
| ERS3422372 |
| ERS3422374 |
| ERS3422376 |
| ERS3422380 |
| ERS3422382 |
| ERS3422386 |
| ERS3422387 |
| ERS3422388 |
| ERS3422390 |
| ERS3422391 |
| ERS3422392 |
| ERS3422393 |
| ERS3422394 |
| ERS3422395 |
| ERS3422396 |
| ERS3422397 |
| ERS3422399 |
| ERS3422401 |
| ERS3422403 |
| ERS3422405 |
| ERS3422407 |
| ERS3422408 |
| ERS3422409 |
| ERS3422410 |
| ERS3422411 |
| ERS3422413 |
| ERS3422414 |
| ERS3422416 |
| ERS3422421 |
| ERS3422425 |
| ERS3422431 |
| ERS3422434 |
| ERS3422437 |
| ERS3422440 |
| ERS3422444 |
| ERS3422445 |
| ERS3422447 |
| ERS3422452 |
| ERS3422456 |
| ERS3422459 |
| ERS3422461 |
| ERS3422463 |
| ERS3422468 |
| ERS3422472 |
| ERS3422473 |
| ERS3422475 |
| ERS3422482 |
| ERS3422484 |
| ERS3422488 |
| ERS3422491 |
| ERS3422497 |
| ERS3422498 |
| ERS3422505 |
| ERS3422507 |
| ERS3422509 |
| ERS3422512 |
| ERS3422514 |
| ERS3422520 |
| ERS3422522 |
| ERS3422527 |
| ERS3422529 |
| ERS3422531 |
| ERS3422534 |
| ERS3422536 |
| ERS3422538 |
| ERS3422541 |
| ERS3422543 |
| ERS3422545 |
| ERS3422548 |
| ERS3422553 |
| ERS3422556 |
| ERS3422564 |
| ERS3422571 |
| ERS3422574 |
| ERS3422577 |
| ERS3422580 |
| ERS3422583 |
| ERS3422588 |
| ERS3422591 |
| ERS3422596 |
| ERS3422602 |
| ERS3422605 |
| ERS3422608 |
| ERS3422611 |
| ERS3422614 |
| ERS3422616 |
| ERS3422622 |
| ERS3422625 |
| ERS3422630 |
| ERS3422632 |
| ERS3422637 |
| ERS3422639 |
| ERS3422642 |
| ERS3422650 |
| ERS3422655 |
| ERS3422657 |
| ERS3422664 |
| ERS3422674 |
| ERS3422678 |
| ERS3422682 |
| ERS3422684 |
| ERS3422691 |
| ERS3422696 |
| ERS3422701 |
| ERS3422704 |
| ERS3422710 |
| ERS3422711 |
| ERS3422716 |
| ERS3422720 |
| ERS3422725 |
| ERS3422728 |
| ERS3422731 |
| ERS3422735 |
| ERS3422737 |
| ERS3422738 |
| ERS3422743 |
| ERS3422748 |
| ERS3422749 |
| ERS3422751 |
| ERS3422755 |
| ERS3422759 |
| ERS3422761 |
| ERS3422766 |
| ERS3422769 |
| ERS3422772 |
| ERS3422775 |
| ERS3422777 |
| ERS3422779 |
| ERS3422783 |
| ERS3422786 |
| ERS3422788 |
| ERS3422791 |
| ERS3422792 |
| ERS3422794 |
| ERS3422796 |
| ERS3422799 |
| ERS3422802 |
| ERS3422804 |
| ERS3422807 |
| ERS3422809 |
| ERS3422811 |
| ERS3422813 |
| ERS3422815 |
| ERS3422817 |
| ERS3422818 |
| ERS3422820 |
| ERS3422823 |
| ERS3422825 |
| ERS3422827 |
| ERS3422829 |
| ERS3422830 |
| ERS3422832 |

**Supplementary Note 2.** List of ENA accession numbers of the Shao et al. and Bäckhed et al. studies for samples used in the analysis of formula impact on ARG load in full-term infants in infancy

| ERR3406255 |
| --- |
| ERR3405874 |
| ERR3405878 |
| ERR3405865 |
| ERR3406251 |
| ERR3405856 |
| ERR3406248 |
| ERR3406050 |
| ERR3406242 |
| ERR3405883 |
| ERR3405991 |
| ERR3405869 |
| ERR3405871 |
| ERR3405872 |
| ERR3405876 |
| ERR3405860 |
| ERR3406048 |
| ERR3405881 |
| ERR3405875 |
| ERR3406260 |
| ERR3406051 |
| ERR3405861 |
| ERR3405989 |
| ERR3405864 |
| ERR3406052 |
| ERR3405990 |
| ERR3405858 |
| ERR3405866 |
| ERR3405859 |
| ERR3405857 |
| ERR3406247 |
| ERR3405993 |
| ERR3405873 |
| ERR3406193 |
| ERR3405868 |
| ERR3406244 |
| ERR3405870 |
| ERR3405911 |
| ERR3405992 |
| ERR3405879 |
| ERR3405877 |
| ERR3405867 |
| ERR3406266 |
| ERR3406250 |
| ERR3406087 |
| ERR3406257 |
| ERR3406259 |
| ERR3406254 |
| ERR3406263 |
| ERR3406265 |
| ERR3406279 |
| ERR3406283 |
| ERR3406105 |
| ERR3405930 |
| ERR3406053 |
| ERR3406055 |
| ERR3406194 |
| ERR3406017 |
| ERR3406056 |
| ERR3406195 |
| ERR3405932 |
| ERR3405926 |
| ERR3405999 |
| ERR3405925 |
| ERR3405902 |
| ERR3406246 |
| ERR3406101 |
| ERR3406024 |
| ERR3406021 |
| ERR3405997 |
| ERR3406006 |
| ERR3405916 |
| ERR3405900 |
| ERR3406196 |
| ERR3406026 |
| ERR3405936 |
| ERR3405884 |
| ERR3405998 |
| ERR3405913 |
| ERR3406016 |
| ERR3405893 |
| ERR3406067 |
| ERR3405929 |
| ERR3405934 |
| ERR3405907 |
| ERR3406197 |
| ERR3406029 |
| ERR3406004 |
| ERR3405894 |
| ERR3405885 |
| ERR3406057 |
| ERR3405931 |
| ERR3406073 |
| ERR3406198 |
| ERR3406011 |
| ERR3406012 |
| ERR3405908 |
| ERR3406253 |
| ERR3406199 |
| ERR3405899 |
| ERR3405892 |
| ERR3406025 |
| ERR3405912 |
| ERR3405896 |
| ERR3406267 |
| ERR3406200 |
| ERR3406008 |
| ERR3406098 |
| ERR3406018 |
| ERR3406096 |
| ERR3406037 |
| ERR3406102 |
| ERR3406001 |
| ERR3405914 |
| ERR3405906 |
| ERR3405888 |
| ERR3405933 |
| ERR3405890 |
| ERR3406015 |
| ERR3406000 |
| ERR3405942 |
| ERR3405995 |
| ERR3405905 |
| ERR3406201 |
| ERR3406202 |
| ERR3405909 |
| ERR3406203 |
| ERR3406023 |
| ERR3406258 |
| ERR3405910 |
| ERR3406204 |
| ERR3406031 |
| ERR3406205 |
| ERR3405898 |
| ERR3406108 |
| ERR3405889 |
| ERR3405939 |
| ERR3406003 |
| ERR3405935 |
| ERR3406002 |
| ERR3406068 |
| ERR3405903 |
| ERR3406074 |
| ERR3406208 |
| ERR3406302 |
| ERR3405943 |
| ERR3405923 |
| ERR3406080 |
| ERR3406209 |
| ERR3406210 |
| ERR3406261 |
| ERR3406264 |
| ERR3406256 |
| ERR3406262 |
| ERR3406093 |
| ERR3406082 |
| ERR3406284 |
| ERR3406269 |
| ERR3406076 |
| ERR3406088 |
| ERR3406282 |
| ERR3406280 |
| ERR3406070 |
| ERR3406276 |
| ERR3406273 |
| ERR3406301 |
| ERR3406291 |
| ERR3406286 |
| ERR3406270 |
| ERR3406289 |
| ERR3406277 |
| ERR3406272 |
| ERR3406290 |
| ERR3406293 |
| ERR3406271 |
| ERR3406287 |
| ERR3406099 |
| ERR3406285 |
| ERR3406299 |
| ERR3406292 |
| ERR3406288 |
| ERR3406294 |
| ERR3406300 |
| ERR3406295 |
| ERR3406211 |
| ERR3406081 |
| ERR3406028 |
| ERR3406212 |
| ERR3406252 |
| ERR3406213 |
| ERR3405918 |
| ERR3406215 |
| ERR3406216 |
| ERR3406058 |
| ERR3406022 |
| ERR3406217 |
| ERR3406218 |
| ERR3406035 |
| ERR3405924 |
| ERR3406219 |
| ERR3406220 |
| ERR3406092 |
| ERR3406221 |
| ERR3406222 |
| ERR3406030 |
| ERR3406223 |
| ERR3406027 |
| ERR3406224 |
| ERR3406059 |
| ERR3406060 |
| ERR3406225 |
| ERR3406226 |
| ERR3406227 |
| ERR3406097 |
| ERR3406036 |
| ERR3406228 |
| ERR3406229 |
| ERR3406298 |
| ERR3406249 |
| ERR3405927 |
| ERR3406231 |
| ERR3406019 |
| ERR3405919 |
| ERR3406232 |
| ERR3406233 |
| ERR3406038 |
| ERR3405937 |
| ERR3406234 |
| ERR3406243 |
| ERR3406235 |
| ERR3405941 |
| ERR3406236 |
| ERR3406075 |
| ERR3406237 |
| ERR3406238 |
| ERR3405938 |
| ERR3406239 |
| ERR3405920 |
| ERR3406103 |
| ERR3406240 |
| ERR3406020 |
| ERR3406241 |
| ERR3406063 |
| ERR3406064 |
| ERR3406268 |
| ERR3405921 |
| ERR525688 |
| ERR525689 |
| ERR525700 |
| ERR525701 |
| ERR525703 |
| ERR525717 |
| ERR525722 |
| ERR525730 |
| ERR525734 |
| ERR525738 |
| ERR525743 |
| ERR525760 |
| ERR525768 |
| ERR525772 |
| ERR525776 |
| ERR525780 |
| ERR525784 |
| ERR525796 |
| ERR525801 |
| ERR525806 |
| ERR525816 |
| ERR525820 |
| ERR525828 |
| ERR525840 |
| ERR525844 |
| ERR525848 |
| ERR525856 |
| ERR525860 |
| ERR525864 |
| ERR525881 |
| ERR525886 |
| ERR525896 |
| ERR525898 |
| ERR525904 |
| ERR525908 |
| ERR525912 |
| ERR525916 |
| ERR525920 |
| ERR525925 |
| ERR525932 |
| ERR525936 |
| ERR525940 |
| ERR525945 |
| ERR525956 |
| ERR525960 |
| ERR525964 |
| ERR525968 |
| ERR525972 |
| ERR525976 |
| ERR525980 |
| ERR525984 |
| ERR525988 |
| ERR526000 |
| ERR526008 |
| ERR526012 |
| ERR526020 |
| ERR526028 |
| ERR526032 |
| ERR526036 |
| ERR526040 |
| ERR526044 |
| ERR526048 |
| ERR526052 |
| ERR526060 |
| ERR526068 |
| ERR526070 |
| ERR526076 |
| ERR526080 |
| ERR526084 |
